# Early dynamics of multimorbidity in 17.4 million people in Spain: onset patterns and prognostic value

**DOI:** 10.1093/eurpub/ckag067

**Published:** 2026-04-27

**Authors:** Ignatios Ioakeim-Skoufa, Jorge Vicente-Romero, Francisca González-Rubio, Mercedes Aza-Pascual-Salcedo, Clara Laguna-Berna, Ermengol Sempere-Verdú, Caterina Vicens-Caldentey, Vicente Palop-Larrea, María Pilar Arroyo-Aniés, Óscar Esteban-Jiménez, Ramón Orueta-Sánchez, Isabel Barrio-Díez, Pilar Marín-Murillo, Sallie-Anne Pearson, Carmen Gómez-Vaquero, Antonio Gimeno-Miguel, Miguel Ángel Hernández-Rodríguez

**Affiliations:** Faculty of Medicine and Health Sciences, Universitat de Barcelona (UB), Barcelona, Spain; Division of Health Data and Digitalisation, Department of Drug Statistics, Norwegian Institute of Public Health, Oslo, Norway; EpiChron Research Group on Chronic Diseases, Aragon Health Sciences Institute (IACS), Aragon Health Research Institute (IIS Aragón), Miguel Servet University Hospital, Zaragoza, Spain; Department of Pharmacology, Physiology and Legal and Forensic Medicine, Faculty of Medicine, University of Zaragoza, Zaragoza, Spain; Spanish Society of Family and Community Medicine (semFYC), Drug Utilisation Work Group, Barcelona, Spain; Research Network on Chronicity, Primary Care and Health Promotion (RICAPPS), Institute of Health Carlos III (ISCIII), Madrid, Spain; Department of Pharmacology, Physiology and Legal and Forensic Medicine, Faculty of Medicine, University of Zaragoza, Zaragoza, Spain; EpiChron Research Group on Chronic Diseases, Aragon Health Sciences Institute (IACS), Aragon Health Research Institute (IIS Aragón), Miguel Servet University Hospital, Zaragoza, Spain; Spanish Society of Family and Community Medicine (semFYC), Drug Utilisation Work Group, Barcelona, Spain; EpiChron Research Group on Chronic Diseases, Aragon Health Sciences Institute (IACS), Aragon Health Research Institute (IIS Aragón), Miguel Servet University Hospital, Zaragoza, Spain; Research Network on Chronicity, Primary Care and Health Promotion (RICAPPS), Institute of Health Carlos III (ISCIII), Madrid, Spain; Aragon Health Service (SALUD), Zaragoza, Spain; EpiChron Research Group on Chronic Diseases, Aragon Health Sciences Institute (IACS), Aragon Health Research Institute (IIS Aragón), Miguel Servet University Hospital, Zaragoza, Spain; Research Network on Chronicity, Primary Care and Health Promotion (RICAPPS), Institute of Health Carlos III (ISCIII), Madrid, Spain; Spanish Society of Family and Community Medicine (semFYC), Drug Utilisation Work Group, Barcelona, Spain; Paterna Primary Care Centre, Conselleria de Sanitat Universal i Salut Pública, Valencia, Spain; Spanish Society of Family and Community Medicine (semFYC), Drug Utilisation Work Group, Barcelona, Spain; Son Serra-La Vileta Primary Care Centre, Servicio de Salud de las Islas Baleares Ib-salut, Palma, Spain; Institut d‘Investigació Sanitaria Illes Balears (IDISBA), Palma de Mallorca, Spain; Spanish Society of Family and Community Medicine (semFYC), Drug Utilisation Work Group, Barcelona, Spain; Hospital IMSKE (Instituto Musculo Esquelético Europeo), Valencia, Spain; Spanish Society of Family and Community Medicine (semFYC), Drug Utilisation Work Group, Barcelona, Spain; Spanish Society of Family and Community Medicine (semFYC), Drug Utilisation Work Group, Barcelona, Spain; Sádaba Primary Care Centre, Zaragoza, Spain; Spanish Society of Family and Community Medicine (semFYC), Drug Utilisation Work Group, Barcelona, Spain; Sillería Primary Care Centre, Toledo, Spain; Spanish Society of Family and Community Medicine (semFYC), Drug Utilisation Work Group, Barcelona, Spain; Spanish Society of Family and Community Medicine (semFYC), Drug Utilisation Work Group, Barcelona, Spain; Medicines Intelligence Research Program, School of Population Health, University of New South Wales, Sydney, Australia; Rheumatology Department, Hospital Universitari de Bellvitge, Barcelona, Spain; EpiChron Research Group on Chronic Diseases, Aragon Health Sciences Institute (IACS), Aragon Health Research Institute (IIS Aragón), Miguel Servet University Hospital, Zaragoza, Spain; Research Network on Chronicity, Primary Care and Health Promotion (RICAPPS), Institute of Health Carlos III (ISCIII), Madrid, Spain; Spanish Society of Family and Community Medicine (semFYC), Drug Utilisation Work Group, Barcelona, Spain; Support and Planning Unit, Directorate of the Canary Islands Health Service, Santa Cruz de Tenerife, Spain

## Abstract

Multimorbidity, the coexistence of two or more chronic diseases, has become a defining challenge for ageing societies. Yet evidence on when and how chronic diseases begin to cluster, and on the prognostic importance of this timing, remains limited. We used nationwide primary care electronic health records covering 17.4 million people in Spain to reconstruct the onset and progression of chronic disease over the life course. The first recorded diagnosis was used to trace temporal trajectories of multimorbidity by age and sex. We examined how the first condition, age at diagnosis, and time since that diagnosis were associated with advanced (≥3 diseases), multisystem (≥2 organ systems), and complex multimorbidity (≥3 organ systems), as well as premature mortality (<50 and <65 years) and outcomes related to polypharmacy and high-risk prescribing, using multivariable regression and gradient boosting models. By 2021, more than one in three Spaniards lived with a chronic condition, and over half of adults aged 45 years or older had multimorbidity. Among them, nearly 60% had complex multimorbidity. The time elapsed since the first diagnosis showed the closest link to further accumulation, followed by age at first diagnosis and current age. Later onset, particularly after the mid-50s, was associated with faster decline once disease emerged. The timing of the first chronic condition is a powerful but often overlooked prognostic marker. Public health strategies delaying onset and integrating early management could substantially reduce the long-term burden of multimorbidity in European populations.

## Introduction

In recent decades, advances in medicine and technology have substantially increased life expectancy. The challenge now is to ensure these extra years are lived in good health, with preserved function and meaningful quality of life. A growing concern for clinical practice and public policy is the rising prevalence of people living with multiple chronic conditions—multimorbidity [1, 2]. In primary care, most consultations involve patients with multimorbidity, and while prevalence increases with age, the absolute number of people under 65 with multimorbidity exceeds those aged over 65 [3–6].

For more than 50 years, multimorbidity has remained a concern in both medicine and public health, as caring for people with several chronic diseases challenges even the most patient-centred and well-coordinated systems [7]. The coexistence of multiple diseases is linked to functional decline, inappropriate polypharmacy, exposure to high-risk medicines, and worse clinical outcomes, including higher mortality [8–10]. Crucially, effects are not merely additive: interactions among diseases, medicines, and life circumstances compound burden for patients, caregivers, and health and social care systems, and widen disparities when guidelines are tailored to single diseases [11].

Early characterisations of multimorbidity were cross-sectional. In Scotland, Barnett and colleagues reported that 23.2% of adults had two or more chronic conditions, occurring earlier and more frequent in deprived groups [4]. In England, Head and colleagues showed widening socioeconomic inequalities in incident and prevalent multimorbidity from 2004 to 2019 [6]. More recently, longitudinal research has begun to show how multimorbidity develops and progresses. A scoping review by Cezard and colleagues synthesised diverse approaches and found that older age, socioeconomic deprivation, overweight, and unhealthy behaviours consistently predict earlier onset and faster progression [12]. Large population-based studies add depth to this picture. Linked records from Wales indicate faster accumulation of chronic conditions in the most deprived communities [13], whereas Spanish cohort data among older adults point to both stable and evolving clusters of diseases over time [14].

Additional longitudinal work has emphasised the prognostic implications of the timing and pace of multimorbidity. When multimorbidity begins before age 45, risks of disability, frailty, and adverse social outcomes are higher [15]. Additionally, the emergence of multimorbidity during midlife is strongly associated with a higher risk of developing dementia later on, underscoring the necessity for preventive measures starting from the very first diagnosis of a chronic disease [16]. Faster accumulation relates to declines in functional and cognitive abilities and to increased mortality [17]. Despite this, evidence remains limited on how chronic conditions first develop and accumulate across the lifespan in the general population, and whether features of the first recorded condition (its identity, the age at first recorded diagnosis, and the time elapsed since that diagnosis) have prognostic value for clinical complexity, prescribing risk, or premature death.

To address these gaps, we analysed health records from 17 423 343 people in Spain to map the most frequent clinical trajectories of multimorbidity and to examine whether early indicators (such as the type of initial condition, the age at first recorded diagnosis, and the time elapsed since that diagnosis) were associated with subsequent advanced or complex multimorbidity, polypharmacy, exposure to high-risk medicines, and premature mortality. Our aim was to provide a population-level descriptive mapping with exploratory prognostic insights, to inform prevention and service planning rather than causal inference.

## Methods

### Study design and population

We conducted a cross-sectional, population-based study with retrospective reconstruction of diagnostic trajectories, using data from the Pharmacoepidemiological Research Database for Public Health Systems (BIFAP; https://bifap.aemps.es). The analysis included all people registered in BIFAP as of 2021, incorporating the full retrospective diagnostic history available in primary care electronic health records. This design allowed us to describe temporal patterns of chronic disease accumulation at the population level, offering a panoramic, descriptive view of multimorbidity across the life course in Spain.

BIFAP is one of Europe’s largest real-world health databases, comprising anonymised electronic health records routinely recorded by healthcare professionals in the Spanish National Health System. It is a non-profit, longitudinal database administered by the Spanish Agency for Medicines and Medical Devices (AEMPS) and supported by Autonomous Communities through collaboration agreements. The database includes demographic, clinical, and prescription data, hospitalisations, laboratory results, and lifestyle information. BIFAP contributes to the European Medicines Agency’s DARWIN EU initiative for real-world evidence generation [18]. A detailed description is available elsewhere [19].

### Variables

Sociodemographic variables included age and sex. Chronic diseases were identified using the full list of 18 conditions from the *Strategy for Addressing Chronicity in the Spanish National Health System* [20], an official framework developed by the Ministry of Health in collaboration with national scientific and professional associations. All 18 chronic conditions defined in the national strategy were included without modification to ensure alignment with Spain’s official framework for chronic disease surveillance and comparability with other studies using the same standard. These conditions, listed in Table S1 with corresponding SNOMED-CT codes, encompass major cardiometabolic, neurologic, respiratory, musculoskeletal, oncologic, hepatologic, renal, immune deficiency, and mental health disorders. Within the mental health domain, depression was analysed as a separate condition, while other mental health disorders comprised chronic psychiatric diagnoses other than depression, including anxiety and stress-related disorders, psychotic and schizoaffective disorders, bipolar disorder, substance use disorders, eating disorders, and intellectual disability.

For each chronic condition, we identified the age at first recorded diagnosis, defined as the earliest entry in the electronic health record, acknowledging that this reflects first documentation rather than biological onset. We then calculated the interval from that diagnosis to the end of follow-up in 2021. All-cause mortality was recorded, and premature death was defined as occurring before ages 50 and 65.

People with at least one of the 18 chronic conditions were classified as having a chronic condition. Multimorbidity was defined as ≥2 chronic conditions and advanced multimorbidity as ≥3. To provide a more stringent, system-based perspective, the 18 conditions were grouped into nine organ-system profiles (cardiometabolic, renal, respiratory, neurologic, musculoskeletal, mental health, oncologic, hepatologic, and immune deficiency; Table S2). Multisystem multimorbidity was defined as conditions from ≥2 organ systems, and complex multimorbidity as conditions from ≥3. This approach identifies people whose conditions span multiple physiological systems, capturing complexity beyond simple disease counts.

Chronic medicine use was assessed using the Anatomical Therapeutic Chemical (ATC) classification (2024 edition). Chronic use was defined as ≥6 months of continuous treatment (≤30-day gap) within the same ATC 4th level, excluding medical devices and group V products. For group D, only codes meeting chronicity criteria were included (Table S3). For each person, we selected the 6-month period in 2021 with the highest number of concurrent chronic medicines; if tied, the one with longest intended duration. High-risk medicines were identified according to the Ministry of Health’s *List of High-Risk Medicines for Chronic Patients* (MARC) (Table S4) [21], and anticholinergic burden was calculated using validated scores (Table S5) [22]. Polypharmacy was defined as ≥5 chronic medicines and excessive polypharmacy as ≥10.

### Temporal trajectories of multimorbidity onset

We reconstructed the chronological sequence of first diagnoses for all chronic conditions recorded in each person’s history. For people entering chronic care with a single condition, we described the most frequent trajectories of multimorbidity onset, stratified by age and sex, and examined subsequent development of additional conditions, polypharmacy, high-risk medicine use, and anticholinergic exposure.

### Statistical analysis

We developed multivariable logistic regression models to predict premature death (before ages 65 and 50). To address class imbalance, we applied the Synthetic Minority Over-sampling Technique (SMOTE) [23]. We imputed missing values for age at first recorded diagnosis using multiple imputation. We standardised all continuous variables before modelling.

We also used gradient boosting (XGBoost) to predict advanced, multisystem, and complex multimorbidity, as well as polypharmacy, high-risk medicine use, and anticholinergic exposure. Models were trained using 5-fold cross-validation stratified by outcome prevalence to ensure balanced evaluation and mitigate overfitting. Hyperparameters were optimised and low-contribution variables excluded to enhance model generalisability. We evaluated performance using accuracy, precision, recall, F1 score, and area under the receiver operating characteristic curve (AUC). We assessed feature importance for each model.

Given the association between age at first recorded diagnosis and adverse outcomes, we used ROC curve analysis and the Youden index to identify optimal age thresholds for each outcome [24].

Additional technical specifications are available in the Supplementary Material (p. 11).

### Ethical considerations

The study was approved by the BIFAP Scientific Committee (11_2022) and the Ethics Committee of the Complejo Hospitalario Universitario de Canarias (CHUNSC_2022_89). It complied with the Declaration of Helsinki, national and EU data protection regulations, and the STROBE reporting guidelines [25].

## Results

### Clinical characteristics of the population

The study included 17 423 343 people (51.28% women). Chronic diseases were identified in 6 738 993 (38.68%). Table 1 summarises clinical characteristics, with breakdowns by sex and age in Table S6. The burden of chronic disease increased sharply with age, from around 12% in children to nearly 90% among those aged 80 years or older, and was consistently higher in women.

**Table 1.** Clinical characteristics of the study population by sex and age group

	All	Women	Men
**Total population**			
Population	17 423 343 (100.00)	8 934 222 (51.28)	8 489 121 (48.72)
Age, mean (SD^a^)	42.88 (23.15)	44.09 (23.60)	41.61 (22.59)
≥1 chronic condition	6 738 993 (38.68)	3 680 095 (41.19)	3 058 898 (36.03)
Multimorbidity^b^	2 922 855 (16.78)	1 729 253 (19.36)	1 193 602 (14.06)
Polypharmacy^c^	948 346 (5.44)	540 570 (6.05)	407 776 (4.80)
High-risk medicines^d^	2 227 561 (12.78)	1 236 253 (13.84)	991 308 (11.68)
**People with at least one chronic condition**			
Multimorbidity	2 922 855 (43.37)	1 729 253 (46.99)	1 193 602 (39.02)
Multisystem multimorbidity^e^	2 438 114 (36.18)	1 527 602 (41.51)	910 512 (29.77)
Complex multimorbidity^f^	765 211 (11.35)	530 359 (14.41)	234 852 (7.68)
Polypharmacy	851 776 (12.64)	485 946 (13.20)	365 830 (11.96)
Excessive polypharmacy^g^	107 287 (1.59)	65 252 (1.77)	42 035 (1.37)
High-risk medicines	1 795 320 (26.64)	996 880 (27.09)	798 440 (26.10)
Anticholinergic activity medicines^h^	830 471 (12.32)	554 018 (15.05)	276 453 (9.04)
High anticholinergic burden^i^	133 732 (1.98)	90 426 (2.46)	43 306 (1.42)

Data are presented as number of people, with percentages in parentheses, unless otherwise specified.

a *SD* = standard deviation.

b Multimorbidity: ≥2 chronic conditions.

c Polypharmacy: ≥5 concurrent medicines for ≥6 months (≤30-day gaps).

d High-risk medicine: ≥1 medicine classified as high-risk for chronic patients.

e Multisystem multimorbidity: Conditions affecting ≥2 organ systems.

f Complex multimorbidity: Conditions affecting ≥3 organ systems.

g Excessive polypharmacy ≥10 concurrent medicines for ≥6 months (≤30-day gaps).

h Anticholinergic activity: ≥1 medicine with known anticholinergic properties.

i High anticholinergic burden: Anticholinergic score ≥3.

**Figure 1.**
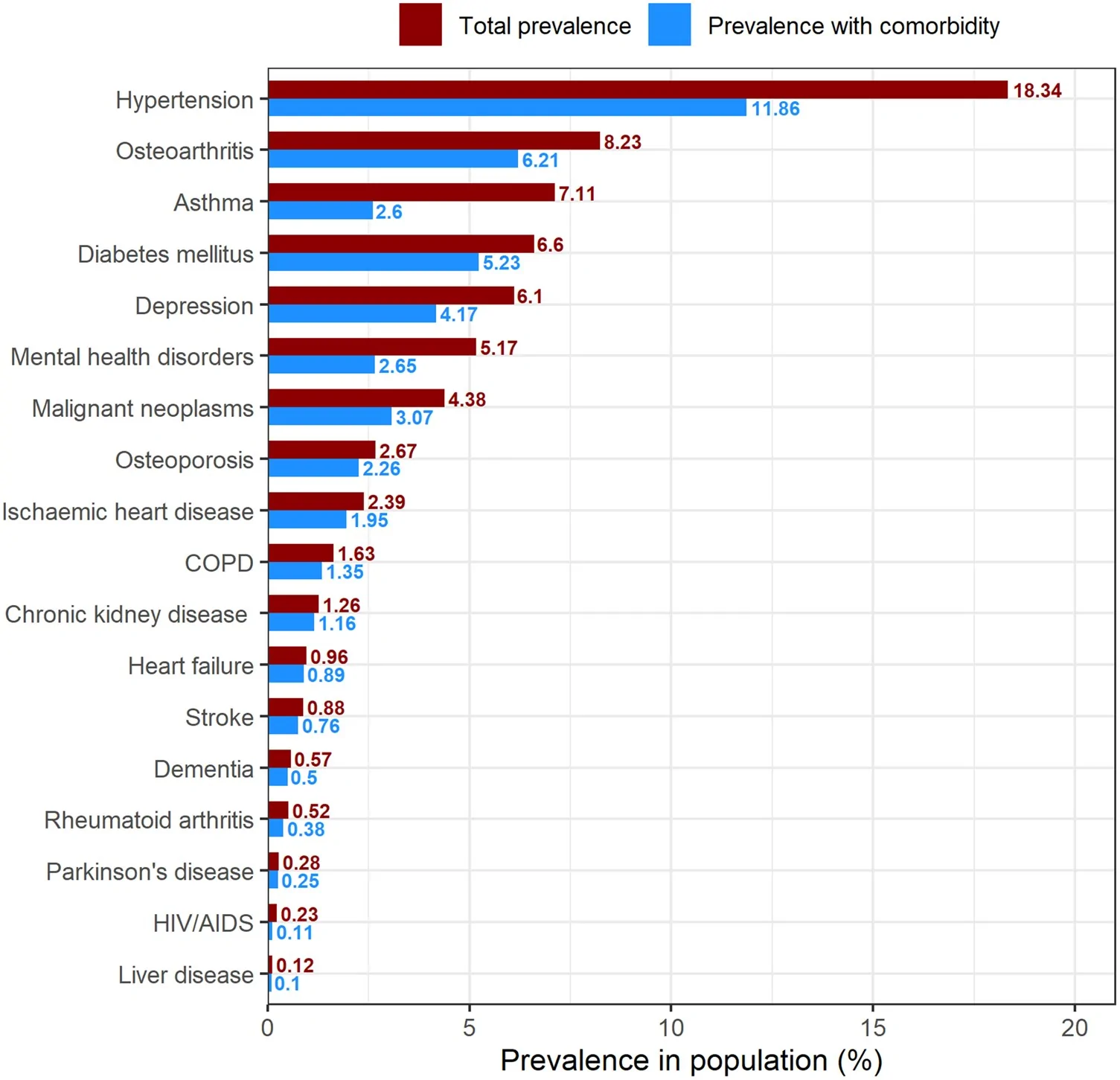
Prevalence of chronic conditions and distribution of comorbidity status. For each condition, the bars show its prevalence in the general population (*n* = 17 423 343). The red bar indicates the total prevalence (regardless of whether it occurs alone or with other conditions), and the blue bar shows the prevalence of individuals who have that condition in combination with at least one other chronic disease (i.e. with comorbidity). Mental health disorders refer to chronic mental health conditions other than depression. *COPD* = chronic obstructive pulmonary disease. *HIV/AIDS* = human immunodeficiency virus infection and acquired immune deficiency syndrome.

Hypertension was the most common chronic condition, affecting 18.34% (3 194 597 people), followed by osteoarthritis and asthma. Diabetes, depression, and other mental health disorders each affected over 5% of the population (Fig. 1; Table S7). Asthma was most often isolated, whereas most other conditions, particularly cardiovascular and metabolic diseases, coexisted with others.

Multimorbidity (≥2 conditions) affected 2 930 447 people (16.82% of the total population). Over half of adults aged ≥45 years with chronic conditions (53.26%; 2 692 810 people) experienced multimorbidity, and over one in four used at least one high-risk medicine.

Multisystem multimorbidity (≥2 organ systems affected) affected 13.99% of the total population (2 438 114 people), rising to 44.22% among chronic patients aged ≥45 years and 57.25% among those aged ≥65 years. Most people with multimorbidity (83.20%) met criteria for multisystem multimorbidity. Complex multimorbidity (≥3 organ systems) affected 4.39% of the total population (765 211 people), 69.31% of whom were women.

A total of 2 227 561 people (12.78% of the general population) were supplied with a high-risk medicine, reaching 46.60% among those aged ≥80 years. Benzodiazepines were most frequent in women from age 15 onwards. Other common high-risk medicines included platelet aggregation inhibitors, β-blockers, sulfonylureas, opioids, and propionic acid derivatives (Table S8).

### Temporal trajectories of multimorbidity onset

Among chronic patients, 6 502 595 people (96.49%) had a single chronic disease at initial diagnosis, whereas 236 398 (3.51%) had two or more conditions recorded on the same date. We focused trajectory analyses on people with a single initial diagnosis to ensure clear and interpretable trajectories. Nonetheless, the most frequent combinations among those with multiple initial diagnoses are summarised in Table S9.

Hypertension was the most frequent first diagnosis (31.69%), followed by asthma (15.48%) and osteoarthritis (10.01%) (Fig. S1; Table S10). Among the population under 45 years of age, nine out of ten people who transitioned to chronicity with a single disease had either asthma, mental health disorders, depression, hypertension, or malignant neoplasms as their initial diagnosis. Among adults aged ≥45, diabetes and osteoporosis were also frequent. Additionally, we observed distinct clinical trajectories for the onset of multimorbidity between women and men, as well as across different age groups. Tables S11 and S12 illustrate the most common trajectories of multimorbidity onset for women and men, respectively, stratified by age group. Further details on common multimorbidity-onset trajectories linked to each of the 18 chronic conditions, broken down by sex, are shown in Tables S13 and S14.

In the paediatric population, initial diagnoses were most often asthma, mental health disorders, or malignant neoplasms. In those aged 15–44 years, asthma predominated, followed by mental health disorders. From age 45 years onwards, hypertension was the most frequent first diagnosis and the most common second condition. In the 45–64 years group, depression and other mental health disorders were frequent first diagnoses, particularly in women. In men aged ≥45 years, diabetes ranked second after hypertension and often co-occurred with it. Osteoarthritis was a common first or second diagnosis in adults ≥45 years, while osteoporosis occurred mainly in women aged ≥65 years.

Trajectories involving diabetes as either the first or second condition showed a higher prevalence of high-risk medicine use. Medicines with anticholinergic activity were more common in trajectories including depression, heart failure, chronic kidney disease, dementia, or Parkinson’s disease, especially among women.

### Premature mortality

In 2021, 145 747 people died, including 4567 before age 50 and 19 409 before 65. Mortality was higher in men across all groups, with the largest relative difference observed for premature deaths. Using multivariable logistic regression, we explored factors associated with death before ages 50 and 65. A first diagnosis of malignant neoplasm showed the strongest association with premature mortality (OR 2.73 for <65 years; 4.56 for <50). Mortality also increased with multimorbidity involving a greater number of organ systems (OR 1.99 and 3.48, respectively; Table 2).

**Table 2.** Top predictors of premature mortality before ages 50 and 65, with log-odds estimates from logistic regression models

Mortality <50 years	Mortality <65 years
First diagnosis: Malignant neoplasms (1.52)	First diagnosis: Malignant neoplasms (1.00)
Number of different organ systems^a^ (1.25)	Age at first recorded diagnosis (0.76)
First diagnosis: Other mental health disorders (0.92)	Number of different organ systems^a^ (0.69)
Multimorbidity (0.88)	First diagnosis: Hypertension (0.56)
First diagnosis: Hypertension (0.87)	Multimorbidity (0.55)
First diagnosis: Depression (0.67)	First diagnosis: Diabetes (0.44)
Total number of diseases (0.62)	First diagnosis: Other mental health disorders (0.38)
First diagnosis: Asthma (0.60)	First diagnosis: Depression (0.38)
First diagnosis: Diabetes (0.56)	Time since first recorded diagnosis (0.34)
Age at first recorded diagnosis (0.54)	First diagnosis: COPD^b^ (0.33)

Values in parentheses are log-odds estimates from multivariable logistic regression models. Classification accuracy was 0.91 for mortality before age 50 and 0.85 for mortality before age 65. Higher absolute values indicate stronger associations with premature mortality. Variables are listed in order of decreasing importance within each model.

a Refers to the total number of organ systems affected by different chronic conditions, in the context of multimorbidity.

b COPD = chronic obstructive pulmonary disease.

### Predictors of other adverse outcomes

Across all outcomes, the time elapsed since the first diagnosis, the age at that diagnosis, and the person’s current age consistently emerged as the most influential markers. For advanced, multisystem, and complex multimorbidity, as well as for exposure to high-risk or anticholinergic medicines, these temporal factors ranked above disease type or sex in importance. Polypharmacy was chiefly associated with older age, followed by earlier disease onset, higher anticholinergic burden, and the use of high-risk medicines (Table 3).

**Table 3.** Top predictors of advanced, multisystem, and complex multimorbidity, polypharmacy, and supply of high-risk or anticholinergic-activity medicines, with feature importance values from gradient boosting models

Outcome	Predictor	Relative importance (%)
**Advanced multimorbidity^a^**	Time since first recorded diagnosis	26.9
	Age at first recorded diagnosis	21.5
	Age	20.6
	Sex	6.0
	High-risk medicine	4.1
**Multisystem multimorbidity^b^**	Time since first recorded diagnosis	24.1
	Age	20.4
	Age at first recorded diagnosis	9.1
	Sex	6.6
	High-risk medicine	5.4
	First diagnosis: Hypertension	4.7
	Polypharmacy	3.3
	First diagnosis: Diabetes mellitus	3.2
**Complex multimorbidity^c^**	Time since first recorded diagnosis	26.9
	Age at first recorded diagnosis	22.7
	Age	18.9
	Sex	5.7
	High-risk medicine	4.4
**Polypharmacy^d^**	Age	33.0
	Age at first recorded diagnosis	16.6
	High anticholinergic burden	15.4
	High-risk medicine	11.2
	Total number of diseases	10.5
	Time since first recorded diagnosis	6.8
	Number of different organ systems	4.4
**Supply of high-risk medicines^e^**	Time since first recorded diagnosis	21.7
	Age	19.8
	Age at first recorded diagnosis	13.2
	Total number of diseases	9.5
	Sex	6.0
	Number of different organ systems	5.8
	High anticholinergic index	3.8
	First diagnosis: Diabetes mellitus	3.3
**Supply of medicines with anticholinergic activity^f^**	Age	18.9
	Time since first recorded diagnosis	18.5
	Age at first recorded diagnosis	15.1
	Number of different organ systems	8.1
	Total number of diseases	7.8
	Sex	5.1
	Polypharmacy	3.9
	High-risk medicine	3.6

Feature importance values are from gradient boosting models; higher values indicate greater contribution to predictions.

a Advanced multimorbidity: ≥3 chronic conditions.

b Multisystem multimorbidity: Conditions affecting ≥2 organ systems.

c Complex multimorbidity: Conditions affecting ≥3 organ systems.

d Polypharmacy: ≥5 concurrent medicines for ≥6 months (≤30-day gaps).

e High-risk medicine: ≥1 medicine classified as high-risk for chronic patients.

f Anticholinergic activity: ≥1 medicine with known anticholinergic properties.

Model performance was strong across endpoints, with AUCs ranging from 0.89 to 0.96. Accuracy reached 0.92 for polypharmacy and 0.90 for exposure to anticholinergic medicines. Recall exceeded 0.92 for advanced, multisystem, complex multimorbidity, and polypharmacy, although precision was lower for rarer outcomes such as complex multimorbidity (0.16), reflecting a higher share of false positives in that context. Full performance metrics for all models are provided in Table S16.

### Age at first recorded diagnosis thresholds for adverse outcomes

Using ROC curves and the Youden index, optimal thresholds for age at first recorded diagnosis were identified for the six adverse outcomes (Table S17). Cut-off points ranged from 52 years for multisystem multimorbidity and supply of high-risk medicines to 56 years for polypharmacy, with most multimorbidity-related outcomes clustering between 52 and 54 years.

## Discussion

Our analysis of 17.4 million people offers a national picture of how chronic diseases emerge and accumulate. In Spain, more than one in three people already lives with a chronic condition. Among adults aged ≥45 years, over half had multimorbidity, and nearly 60% met criteria for complex multimorbidity involving three or more organ systems. The strongest early markers of progression were temporal features of the first recorded disease (the time since diagnosis and the age at which it occurred) consistently outweighing the influence of disease identity or sex. The single major exception was premature mortality, where a first diagnosis of malignant neoplasm dominated, particularly when several organ systems were already affected. These patterns highlight the need to treat the first chronic diagnosis as a decisive turning point for prevention and integrated care. Our findings should be viewed as population-level descriptive evidence rather than causal inference. By reconstructing diagnostic sequences across the BIFAP population, this study provides a panoramic view of multimorbidity accumulation and its public-health implications [26].

Multimorbidity exposes the limitations of traditional healthcare models that focus on single diseases [4, 5, 27, 28]. As Barnett and colleagues noted, multimorbidity “challenges the single-disease framework” that underpins much of healthcare, research, and medical training [4]. In practice, one disease can increase the risk of another or even lead to new conditions, underscoring the need for collaboration across clinical disciplines and for integrated health and social care [5]. More effective responses include longer, person-centred consultations, multidisciplinary teams that span various specialties, and clinical guidelines that promote holistic management rather than a one-disease-at-a-time approach [3, 4, 27–29]. These reforms are supported by public health initiatives; for example, the recent 10-year health plan for England aims to integrate health and social care services to tackle inequality [30]. This illustrates a broader shift toward upstream, preventive policy.

Mapping how chronic diseases accumulate across the life course helps identify the ‘entry points’ into multimorbidity, sentinel conditions where prevention and coordination can have the greatest impact [31]. In our study, certain conditions acted as sentinel gateways: asthma and mental health disorders in youth, and hypertension, osteoarthritis, and diabetes in mid-to-late life, consistent with previous longitudinal evidence [32].

Time-related characteristics of the first recorded diagnosis (specifically, the number of years since that diagnosis and the age at diagnosis) were the factors most strongly associated with progression to advanced or complex multimorbidity. These temporal markers outweighed both the identity of the initial disease and the patient’s sex. The only major exception was premature mortality (before age 50 or 65), for which a first diagnosis of malignant neoplasm remained the dominant factor, particularly when accompanied by multimorbidity spanning multiple organ systems.

The time elapsed since the first chronic diagnosis emerged as the strongest correlate of progression to advanced, multisystem, and complex multimorbidity. Age at first diagnosis and current age contributed independently but in complementary ways. An early first diagnosis means longer exposure over time, whereas a late first diagnosis often signals lower resilience and a faster accumulation of conditions. These dual mechanisms (prolonged exposure and age-related vulnerability) highlight the importance of both delaying the onset of chronic disease and acting early after the first diagnosis to limit further accumulation. These findings call for public health strategies that anticipate multimorbidity before it becomes entrenched, integrating prevention, early detection, and coordinated management as core elements of chronic disease policy and service design.

Biologically, our findings are consistent with evidence that low-grade systemic inflammation acts as a shared upstream pathway linking many chronic conditions [33–35]. This process is amplified by modifiable risk factors such as obesity, smoking, and physical inactivity, and shaped by broader social stressors that embed inequality into biological vulnerability [36–38]. Recognising these interconnections supports an integrated policy approach combining medical prevention with social and behavioural strategies [28, 30].

These observations reinforce the importance of rethinking prevention. Care pathways should aim to delay or prevent the first chronic condition while ensuring comprehensive, person-centred management once any chronic disease is diagnosed. Early control of shared risk factors, medication review, and monitoring for common comorbidities could help slow disease accumulation and reduce downstream complexity.

Our study has limitations inherent to the use of routine health records. Our estimates rely on diagnoses recorded in routine primary-care electronic health records. As with all studies using coded clinical data, recording completeness and accuracy may vary across conditions. Some chronic diseases may be under-recorded or documented only when clinically prominent. For example, chronic kidney disease is often identified through laboratory parameters rather than explicit diagnostic coding, and osteoarthritis, particularly at certain anatomical sites, may be recorded mainly in more advanced stages. As a result, prevalence may be underestimated for some conditions and the age at onset should be interpreted as the first recorded diagnosis in the electronic health record rather than the biological onset of disease. Because the population comprised people alive and registered in 2021, earlier deaths are under-represented, introducing potential immortal-time bias (people who died before extraction could not contribute trajectories, so early-onset or high-mortality patterns may be underestimated). Consequently, associations are descriptive rather than causal. We used the 18 chronic conditions in Spain’s Strategy for Addressing Chronicity to keep definitions relevant and comparable. Alternative frameworks can yield different groupings, a known challenge in multimorbidity research [20, 28]. To better capture clinical complexity, we introduced multisystem multimorbidity, grouping the 18 conditions into nine organ-system profiles. Socioeconomic circumstances may also influence both the development of multimorbidity and the risk of premature mortality. Individual-level socioeconomic indicators were not available in the present analysis, and residual confounding related to social disadvantage may therefore contribute to some of the observed associations. Finally, the dataset lacked person-level trajectories of behavioural and social risk factors (e.g. smoking, obesity, education), biomarkers or omics, which likely contribute to multimorbidity patterns; future work integrating these dimensions and external settings will be important to refine generalisability and mechanisms. Future research should also examine the pathways linking severe mental disorders and premature mortality suggested by our findings.

Despite these limitations, the nationwide scope and consistent patterns across outcomes support the robustness and public health relevance of our observations. Several implications emerge for prevention and health-system design. First, delaying the onset of the first chronic condition should remain a central goal of life-course prevention [39]. Second, once any chronic condition is diagnosed, health systems should ensure timely coordination of care through comprehensive assessment of medication, mental health, and function, alongside proactive monitoring for common comorbidities. Third, medication-safety measures (such as avoiding high-risk drugs when safer alternatives exist, reducing anticholinergic burden, and promoting structured deprescribing) should be standard in chronic-care pathways. Finally, integrating health and social care provides a realistic route to sustaining prevention while managing complexity [30].

Overall, this study identifies time since first diagnosis as a powerful yet under-recognised prognostic dimension, underscoring the importance of delaying the first chronic condition and promoting integrated care from its onset. Aligning prevention and service delivery with the principles of healthy ageing and equity in European public health policy can help shift attention upstream, to the beginning of the chronic disease process [40]. Our findings advocate for a paradigm shift in how we address chronic disease and multimorbidity. Acting before or at the first diagnosis offers a tangible opportunity to alter long-term trajectories, improve outcomes, and reduce future burdens on patients and health systems.

## Supplementary Material

ckag067_Supplementary_Data

## Data Availability

The datasets analysed during this study are not publicly available because of restrictions imposed by the Pharmacoepidemiological Research Database for Public Health Systems (BIFAP) and enforced by the Spanish Agency of Medicines and Medical Devices (AEMPS) and the corresponding Clinical Research Ethics Committee. Aggregated data by sex and age group are presented within the article and its Supplementary Data. Access to individual-level data is subject to legal and ethical approval and can be requested directly from BIFAP (https://www.bifap.es/). Keypoints The timing of the first chronic disease diagnosis is a crucial yet under-recognised determinant of long-term health outcomes. Delaying the onset of chronic conditions should be a central goal of public health strategies in ageing populations. Early, integrated management after the first diagnosis can slow the progression to complex and multisystem multimorbidity. Routine primary care records can be used to identify people at higher risk of rapid disease accumulation and guide preventive care. Aligning prevention, primary care, and social policy could substantially reduce the future burden of multimorbidity across European health systems.
