# Supplementary material for: Early dynamics of multimorbidity in 17.4 million people in Spain: onset patterns and prognostic value
Source: Eur J Public Health. 2026 Apr 27;36(3):ckag067. doi: 10.1093/eurpub/ckag067 (PMC13117620; doi:10.1093/eurpub/ckag067)
Supplement: ckag067_Supplementary_Data [file ckag067_supplementary_data.docx]

Ioakeim-Skoufa I, et al.

Early dynamics of multimorbidity in 17.4 million people in Spain: onset patterns and prognostic value

**Supplementary Data**

**Index**

Table S1: Complete list of SNOMED CT codes used to define each chronic condition (pp 2-5)

Table S2: Overview of variables included in algorithms and data analysis (pp 6-7)

Table S3: Medicines in ATC Group D (Dermatologicals) considered “chronic” in this study (pp 8)

Table S4: High-risk medicines for chronic patients (pp 9)

Table S5: Anticholinergic activity scores (pp 10)

Statistical analysis – technical details (pp 11)

Table S6: Clinical characteristics of the study population by sex and age group (pp 12-13)

Table S7: Prevalence of chronic conditions in the general population (overall and with comorbidity), stratified by sex and age group (pp 14-15)

Table S8: Prevalence of high-risk medicine use in the general population, stratified by sex and age group (pp 16-19)

Table S9: Most common combinations of chronic diseases at the time of initial diagnosis among people with two or more chronic conditions, stratified by sex and age group (pp 20)

Figure S1: First recorded chronic condition and subsequent comorbidity among incident chronic patients. For each condition, the bars show the prevalence among individuals who entered chronic care with a single initial diagnosis (n = 6 502 595); the red bar indicates the total prevalence of that condition as the first recorded diagnosis (regardless of whether comorbidity developed), and the blue bar shows the prevalence of those who later developed comorbidity. Mental health disorders refer to chronic mental health conditions other than depression. COPD = chronic obstructive pulmonary disease. HIV/AIDS = human immunodeficiency virus infection and acquired immune deficiency syndrome (pp 21)

Table S10: First recorded chronic condition and subsequent trajectory towards multimorbidity, stratified by sex and age group (pp 22-24)

Table S11: Most frequent trajectories of multimorbidity onset in women by age group; progression to ≥3 conditions, polypharmacy, high-risk medicine use, and exposure to medicines with anticholinergic activity (pp 25-26)

Table S12: Most frequent trajectories of multimorbidity onset in men by age group; progression to ≥3 conditions, polypharmacy, high-risk medicine use, and exposure to medicines with anticholinergic activity (pp 27-28)

Table S13: Most common trajectories of multimorbidity onset originating from each of the 18 chronic conditions in women (pp 23-30)

Table S14: Most common trajectories of multimorbidity onset originating from each of the 18 chronic conditions in men (pp 31-32)

Table S15: All-cause mortality in 2021 in the general population and among those with multimorbidity or multisystem multimorbidity, stratified by sex (pp 33)

Table S16: Performance metrics for gradient boosting models predicting advanced, multisystem, and complex multimorbidity, polypharmacy, and supply of high-risk or anticholinergic-activity medicines (pp 34)

Table S17: Optimal age at first recorded diagnosis cut-off points for adverse outcomes, derived from ROC curves using the Youden index (pp 35)

**Table S1: Complete list of SNOMED CT codes used to define each chronic condition.**

| Cardiovascular-metabolic profile | | | | | |
| --- | --- | --- | --- | --- | --- |
|  |  |  |  |  |  |
| **Hypertension** | | | | | |
|  |  |  |  |  |  |
| 10725009 | 194767001 | 266228004 | 44111003 | 56218007 | 70272006 |
| 1201005 | 194785008 | 31992008 | 449759005 | 59621000 | 706882009 |
| 123799005 | 24184005 | 38341003 | 49220004 | 60899001 | 70995007 |
| 129899009 | 250765005 | 38481006 | 50490005 | 64715009 | 827068008 |
| 135840009 | 26014004 | 428163005 | 52698002 | 6962006 | 827069000 |
| 163027005 |  |  |  |  |  |
|  |  |  |  |  |  |
| **Diabetes mellitus** | | | | | |
|  |  |  |  |  |  |
| 44054006 | 311782002 | 770766000 | 81531005 | 5368009 | 368581000119106 |
| 73211009 | 127014009 | 26298008 | 769218003 | 770362001 | 395204000 |
| 46635009 | 25093002 | 713704004 | 445170001 | 1481000119100 | 427134009 |
| 4855003 | 421075007 | 170746002 | 111231000119109 | 190389009 | 860979002 |
| 237599002 | 421893009 | 368581000119106 | 190330002 | 39181008 | 90741000119107 |
| 230572002 | 419100001 | 422088007 | 441656006 | 399871005 | 160670007 |
| 313436004 | 609562003 | 422166005 | 860977000 | 408540003 | 193184006 |
| 127013003 | 770765001 | 735537007 | 421365002 | 420715001 | 201250006 |
| 422014003 | 424736006 | 420270002 | 703138006 | 90781000119102 | 609564002 |
| 390834004 | 237604008 | 422099009 | 860978005 | 11530004 | 707221002 |
| 280137006 | 190447002 | 43959009 | 25412000 | 126534007 | 731000119105 |
| 268519009 | 8801005 | 201252003 | 111556005 | 140391000119101 | 789567007 |
| 59276001 | 312912001 | 761000119102 | 445260006 | 140531000119105 | 1481000119100 |
| 371087003 | 609561005 | 190331003 | 49455004 | 28032008 | 140381000119104 |
| 313435000 | 789542009 | 421750000 | 201251005 | 421468001 | 200687002 |
| 443694000 | 421895002 | 421326000 | 237620003 | 427027005 | 420789003 |
| 420422005 | 420868002 | 769217008 | 444073006 | 721000119107 | 422034002 |
| 34170007 | 422183001 | 422126006 | 368051000119109 | 771000119108 | 609567009 |
| 74627003 | 237633009 | 739681000 | 741000119101 | 816178004 | 713702000 |
| 111552007 | 312904009 | 420279001 | 310505005 | 1521000119100 | 816177009 |
| 312903003 | 421847006 | 236500003 | 31211000119101 | 314902007 |  |
|  |  |  |  |  |  |
| **Heart failure** | | | | | |
|  |  |  |  |  |  |
| 84114007 | 83291003 | 79955004 | 56675007 | 443343001 | 443344007 |
| 42343007 | 71892000 | 410431009 | 49584005 | 446221000 | 10633002 |
| 55827005 | 418304008 | 89792004 | 153941000119100 | 5148006 | 88805009 |
| 85232009 | 102558002 | 92506005 | 364006 | 443253003 | 194781004 |
| 367363000 | 266249003 | 417996009 | 360371003 | 271809000 | 426263006 |
| 424372002 | 82608003 | 441530006 | 443254009 | 206586007 | 698296002 |
| 40541001 | 35304003 | 441481004 | 442304009 | 703272007 | 82014009 |
| 195111005 | 67599009 | 46113002 | 194767001 | 195112003 | 82523003 |
| 48447003 |  |  |  |  |  |
|  |  |  |  |  |  |
| **Ischaemic heart disease** | | | | | |
|  |  |  |  |  |  |
| 194828000 | 53741008 | 81953000 | 233817007 | 443502000 | 28931004 |
| 57054005 | 414024009 | 386138005 | 398274000 | 194842008 | 65340007 |
| 426396005 | 414795007 | 429639007 | 41339005 | 15960061000119102 | 732230001 |
| 414545008 | 233819005 | 307140009 | 426856002 | 371803003 | 442421004 |
| 22298006 | 394659003 | 36969009 | 50570003 | 90487008 | 194823009 |
| 413844008 | 233823002 | 713405002 | 413439005 | 25106000 | 10190003 |
| 413838009 | 401303003 | 401314000 | 84537008 | 59062007 | 14201006 |
| 225566008 | 63739005 | 35304003 | 194843003 | 78741000119103 | 15256002 |
| 4557003 | 33511000122101 | 233845001 | 23687008 | 15960141000119102 | 29819009 |
| 300995000 | 87343002 | 29899005 | 14323007 | 59021001 | 42531007 |
| 1755008 | 232717009 | 81266008 | 92517006 | 46109009 | 47338008 |
| 371030007 | 73795002 | 314116003 |  |  |  |

(Table S1 continues on next page)

**Table S1: Complete list of SNOMED CT codes used to define each chronic condition.**

(continued from previous page)

| **Stroke** | | | | | |
| --- | --- | --- | --- | --- | --- |
|  |  |  |  |  |  |
| 230690007 | 62914000 | 281864001 | 270907008 | 75038005 | 195168007 |
| 422504002 | 274100004 | 82999001 | 449020009 | 149821000119103 | 143521000119103 |
| 432504007 | 195190007 | 9901000119100 | 95453001 | 738779002 | 23276006 |
| 230698000 | 275526006 | 1131000119105 | 301765007 | 301764006 | 34181000119102 |
| 371040005 | 371041009 | 65312002 | 450425005 | 230719004 | 195189003 |
| 230706003 | 1386000 | 427296003 | 373606000 | 428268007 | 34191000119104 |
| 21454007 | 95460007 | 450362008 | 431266005 | 137991000119103 | 125081000119106 |
|  |  |  |  |  |  |
| Renal profile | | | | | |
|  |  |  |  |  |  |
| **Chronic kidney disease** | | | | | |
|  |  |  |  |  |  |
| 723190009 | 302497006 | 310647000 | 426340003 | 423062001 | 417038009 |
| 433144002 | 108241001 | 78544004 | 718308002 | 450866001 | 717738008 |
| 90688005 | 104931000119100 | 79827002 | 718331002 | 238318009 | 19765000 |
| 700378005 | 71192002 | 473034005 | 408667000 | 385970002 | 236434000 |
| 700379002 | 433146000 | 49220004 | 443596009 | 440084005 | 251859005 |
| 709044004 | 236433006 | 443143006 | 39482009 | 473397008 | 276883000 |
| 431857002 | 429075005 | 406168002 | 430958003 | 63421002 | 292760002 |
| 431856006 | 714152005 | 225892009 | 431028002 | 698937002 | 397849009 |
| 431855005 | 46177005 | 385971003 | 69380006 | 713724000 | 736919006 |
| 265764009 | 8501000119104 |  |  |  |  |
|  |  |  |  |  |  |
| Respiratory profile | | | | | |
|  |  |  |  |  |  |
| **Chronic obstructive pulmonary disease (COPD)** | | | | | |
|  |  |  |  |  |  |
| 13645005 | 185086009 | 313296004 | 195949008 | 52571006 | 135836000 |
| 63480004 | 313297008 | 10692761000119108 | 106001000119101 | 68328006 | 31898008 |
| 195951007 | 61937009 | 313299006 | 195957006 | 285381006 | 4981000 |
| 87433001 | 293241000119100 | 74417001 | 196001008 |  |  |
|  |  |  |  |  |  |
| **Asthma** | | | | | |
|  |  |  |  |  |  |
| 195967001 | 405720007 | 708093000 | 708095007 | 426656000 | 195949008 |
| 405944004 | 31387002 | 370218001 | 708094006 | 370221004 | 57607007 |
| 233683003 | 233678006 | 426979002 | 71892000 | 708096008 | 233679003 |
| 389145006 | 427603009 | 427295004 | 370219009 | 708090002 | 63088003 |
| 281239006 | 409663006 | 370220003 | 55570000 | 445427006 | 708038006 |
| 266361008 | 427679007 |  |  |  |  |
|  |  |  |  |  |  |
| Neurologic profile | | | | | |
|  |  |  |  |  |  |
| **Dementia** | | | | | |
|  |  |  |  |  |  |
| 26929004 | 80098002 | 56267009 | 191452002 | 1581000119101 | 14070001 |
| 15662003 | 230270009 | 281004 | 191461002 | 191451009 | 421529006 |
| 52448006 | 725898002 | 191459006 | 191455000 | 416975007 | 230284004 |
| 429998004 | 1591000119103 | 425390006 | 191457008 | 191493005 | 22255007 |
| 191519005 | 12348006 | 79341000119107 | 22381000119105 | 792004 | 101421000119107 |
| 191449005 | 371024007 |  |  |  |  |
|  |  |  |  |  |  |
| **Parkinson’s disease** | | | | | |
|  |  |  |  |  |  |
| 49049000 | 64572001 | 44695005 | 230292008 | 308909003 | 230296006 |
| 2798002 |  |  |  |  |  |

(Table S1 continues on next page)

**Table S1: Complete list of SNOMED CT codes used to define each chronic condition.**

(continued from previous page)

| Musculoskeletal profile | | | | | |
| --- | --- | --- | --- | --- | --- |
|  |  |  |  |  |  |
| **Rheumatoid arthritis** | | | | | |
|  |  |  |  |  |  |
| 69896004 | 239791005 | 410798004 | 57160007 | 86219005 | 781206002 |
| 239796000 | 410502007 | 16044751000119106 | 410797009 | 16024431000119108 | 410796000 |
| 239792003 | 201796004 | 84801008 |  |  |  |
|  |  |  |  |  |  |
| **Osteoarthritis** | | | | | |
|  |  |  |  |  |  |
| 239873007 | 201819000 | 428776005 | 323291000119108 | 201837004 | 201852006 |
| 396275006 | 254779008 | 112981000119107 | 82300000 | 33952002 | 16583361000119100 |
| 37895003 | 8847002 | 323321000119100 | 201834006 | 67315001 | 313257005 |
| 41888000 | 268054009 | 239865003 | 201829007 | 318671000119108 | 1074461000119100 |
| 239872002 | 239874001 | 201835007 | 113011000119109 | 239868001 | 1074491000119107 |
| 22193007 | 371598009 | 323301000119109 | 201831003 | 201952005 | 15729841000119100 |
| 58188004 | 274135002 | 323311000119107 | 239878003 | 201855008 | 38850007 |
| 239880009 | 239867006 | 109668000 | 239862000 | 318721000119100 | 299321000 |
| 36186002 | 309246000 | 450521003 | 303041000119104 | 201847001 | 399269003 |
| 840396000 | 239866002 | 267889007 | 112991000119105 |  |  |
|  |  |  |  |  |  |
| **Osteoporosis** | | | | | |
|  |  |  |  |  |  |
| 64859006 | 18040001 | 443165006 | 311891006 | 203453001 | 11311601000119108 |
| 32369003 | 240198002 | 311890007 | 704334005 |  |  |
|  |  |  |  |  |  |
| Mental health profile | | | | | |
|  |  |  |  |  |  |
| **Depression** | | | | | |
|  |  |  |  |  |  |
| 35489007 | 366979004 | 370143000 | 129871000 | 267076002 | 310497006 |
| 87414006 | 300706003 | 48589009 | 111475002 | 87521009 | 442099003 |
| 78667006 |  |  |  |  |  |
|  |  |  |  |  |  |
| **Mental health disorders** | | | | | |
|  |  |  |  |  |  |
| 198288003 | 68890003 | 191627008 | 31216003 | 111484002 | 191531007 |
| 79890006 | 72366004 | 31658008 | 191567000 | 191542003 | 68995007 |
| 6525002 | 26416006 | 83746006 | 31027006 | 191562006 | 63181006 |
| 13746004 | 441704009 | 1376001 | 46206005 | 191559008 | 79866005 |
| 191736004 | 231494001 | 191499009 | 40700009 | 191555002 | 712850003 |
| 58214004 | 191527001 | 26025008 | 7200002 | 71103003 | 712824002 |
| 78004001 | 61152003 | 231496004 | 161468000 | 767631007 | 191571002 |
| 69322001 | 191447007 | 35252006 | 765176007 | 270901009 | 430909002 |
| 64905009 | 86765009 |  |  |  |  |

(Table S1 continues on next page)

**Table S1: Complete list of SNOMED CT codes used to define each chronic condition.**

(continued from previous page)

| Oncologic profile | | | | | |
| --- | --- | --- | --- | --- | --- |
|  |  |  |  |  |  |
| **Malignant neoplasms** | | | | | |
|  |  |  |  |  |  |
| 424413001 | 363514001 | 38713004 | 188606007 | 36087009 | 286887005 |
| 254837009 | 254582000 | 21708004 | 309245001 | 363493006 | 372097009 |
| 399068003 | 128462008 | 413446001 | 285432005 | 363495004 | 408646000 |
| 363406005 | 363449006 | 254829001 | 190092003 | 57513006 | 188241004 |
| 372064008 | 109995007 | 307608006 | 448675008 | 5843004 | 373168002 |
| 781382000 | 92818009 | 307593001 | 25190001 | 188188009 | 443648003 |
| 399326009 | 65877006 | 4079000 | 865954003 | 3898006 | 314998002 |
| 402815007 | 363418001 | 443520009 | 63634009 | 187760008 | 363433009 |
| 399490008 | 45083001 | 836274002 | 93765001 | 188239000 | 94071006 |
| 363354003 | 363486007 | 363508008 | 86616005 | 722688002 | 253000007 |
| 118600007 | 254626006 | 302849000 | 128674003 | 307651005 | 371972005 |
| 2092003 | 41607009 | 363516004 | 269515006 | 447882007 | 713574000 |
| 363478007 | 363402007 | 254611009 | 252989001 | 255073006 | 188250002 |
| 19453003 | 408647009 | 448993007 | 255071008 | 4590003 | 269475001 |
| 447782002 | 91857003 | 372141009 | 449248000 | 128668003 | 363497007 |
| 363358000 | 254915003 | 363353009 | 255028004 | 363379000 | 403902008 |
| 254651007 | 109841003 | 370967009 | 255032005 | 363494000 | 71666005 |
| 63166000 | 301756000 | 126685009 | 449420002 | 93781006 | 118287003 |
| 363349007 | 254878006 | 372142002 | 188154003 | 189251006 | 709830006 |
| 363518003 | 255088001 | 255052006 | 403891008 | 372063002 | 363350007 |
| 118601006 | 28899001 | 94248000 | 363367000 | 726654006 | 363393007 |
| 93984006 | 363410008 | 700423003 | 403892001 | 363409003 | 254637007 |
| 93143009 | 372130007 | 93761005 | 363392002 | 371982006 | 445238008 |
| 109989006 | 91861009 | 363458004 | 408645001 | 188498009 | 709517003 |
| 255108000 | 429033009 | 27090000 | 128627007 | 254632001 | 372138000 |
| 118599009 | 187692001 | 1701000119104 | 373083005 | 36310008 | 443679004 |
| 449096009 | 254900004 | 443719001 | 425178004 | 85956000 | 363415003 |
| 363429002 | 363351006 | 363515000 | 254887002 | 29421000119105 | 363462005 |
| 92814006 | 109385007 | 35868009 | 419052002 | 414628006 | 363463000 |
| 255029007 | 188192002 | 118617000 | 418372008 | 254843006 | 721567004 |
| 302843004 | 41627005 | 363490009 | 112677002 | 363348004 | 94391008 |
| 71298006 | 254838004 | 363414004 | 2985005 | 88400008 | 188244007 |
| 31069005 | 448216007 | 702391001 | 363459007 | 985004 | 705176003 |
| 109994006 | 448952004 | 363375006 | 363475005 | 128843003 | 13583002 |
| 363443007 | 363507003 | 372095001 | 276975007 | 708921005 | 254625005 |
| 118618005 | 269544008 | 187841006 | 12400006 | 12478003 | 187833006 |
| 70921007 | 443961001 | 428281000 | 269533000 | 14799000 | 254828009 |
| 82711006 | 94381002 | 271323007 | 363432004 | 413990004 | 312113007 |
| 254650008 | 94222008 | 126680004 | 405822008 | 703609007 | 314994000 |
| 123845008 | 408643008 | 254645002 | 93720005 | 188243001 | 404041003 |
| 363346000 | 254634000 | 269516007 | 126719004 | 118286007 | 8145008 |
| 93870000 | 432328008 | 45490001 | 372106005 | 254622008 | 94503003 |
| 371973000 | 70179006 | 276803003 | 703655009 | 363412000 | 702392008 |
| 109992005 | 276952000 |  |  |  |  |
|  |  |  |  |  |  |
| Hepatologic profile | | | | | |
|  |  |  |  |  |  |
| **Liver disease** | | | | | |
|  |  |  |  |  |  |
| 19943007 | 328383001 | 76783007 | 31712002 | 1761006 |  |
|  |  |  |  |  |  |
| Immune deficiency profile | | | | | |
|  |  |  |  |  |  |
| **Human immunodeficiency virus infection and acquired immune deficiency syndrome (HIV/AIDS)** | | | | | |
|  |  |  |  |  |  |
| 165816005 | 91947003 | 186706006 | 713531003 | 421529006 | 111880001 |
| 62479008 | 62246005 | 699433000 | 838377003 | 713297001 | 420721002 |
| 86406008 |  |  |  |  |  |
|  |  |  |  |  |  |

**Table S2: Overview of variables included in algorithms and data analysis.**

| Variable | Description |
| --- | --- |
| Sex | Sex (female/male) |
| Age | The age of the patient |
| Number of diseases | The total number of diseases in an individual (hypertension, diabetes mellitus, heart failure, ischaemic heart disease, stroke, chronic kidney disease, chronic obstructive pulmonary disease, asthma, dementia, Parkinson’s disease, rheumatoid arthritis, osteoarthritis, osteoporosis, depression, other mental health problems excl. depression, malignant neoplasms, liver disease, and human immunodeficiency virus infection and acquired immune deficiency syndrome) |
| Age at first recorded diagnosis | Refers to the age at which the first diagnosis was recorded. |
| First diagnosis | The first recorded disease for a patient (referring to the variability across first diagnoses) |
| First diagnosis: hypertension | Arterial hypertension was the first diagnosis |
| First diagnosis: diabetes mellitus | Diabetes mellitus was the first diagnosis |
| First diagnosis: heart failure | Heart failure was the first diagnosis |
| First diagnosis: ischaemic heart disease | Ischaemic heart disease was the first diagnosis |
| First diagnosis: stroke | Stroke was the first diagnosis |
| First diagnosis: chronic kidney disease | Chronic kidney disease was the first diagnosis |
| First diagnosis: chronic obstructive pulmonary disease | Chronic obstructive pulmonary disease was the first diagnosis |
| First diagnosis: asthma | Asthma was the first diagnosis |
| First diagnosis: dementia | Dementia was the first diagnosis |
| First diagnosis: Parkinson’s disease | Parkinson’s disease was the first diagnosis |
| First diagnosis: rheumatoid arthritis | Rheumatoid arthritis was the first diagnosis |
| First diagnosis: osteoarthritis | Osteoarthritis was the first diagnosis |
| First diagnosis: osteoporosis | Osteoporosis was the first diagnosis |
| First diagnosis: depression | Depression was the first diagnosis |
| First diagnosis: mental health problems | A chronic mental health problem (excl. depression) was the first diagnosis |
| First diagnosis: malignant neoplasms | Cancer was the first diagnosis |
| First diagnosis: liver disease | Liver disease was the first diagnosis |
| First diagnosis: human immunodeficiency virus infection and acquired immune deficiency syndrome | Human immunodeficiency virus infection and acquired immune deficiency syndrome was the first diagnosis |
| Multimorbidity | The presence of two or more chronic conditions simultaneously |
| Advanced multimorbidity; stage I | The presence of three or more chronic conditions simultaneously |
| Advanced multimorbidity; stage II | The presence of four or more chronic conditions simultaneously |
| Advanced multimorbidity; stage III | The presence of five or more chronic conditions simultaneously |

(Table S2 continues on next page)

**Table S2: Overview of variables included in algorithms and data analysis.**

(continued from previous page)

| Variable | Description |
| --- | --- |
| Number of systems | The total number of the different systems affected (profiles of chronicity/multimorbidity; see the nine different profiles below) |
| Cardiovascular-metabolic profile | The presence of at least one cardiovascular-metabolic condition (hypertension, diabetes mellitus, heart failure, ischaemic heart disease, stroke) |
| Respiratory profile | The presence of at least one condition respiratory (chronic obstructive pulmonary disease, asthma) |
| Neurologic profile | The presence of at least one neurologic condition (dementia, Parkinson’s disease) |
| Mental health profile | The presence of at least one mental health condition (depression, other chronic mental health problems) |
| Musculoskeletal profile | The presence of at least one musculoskeletal condition (rheumatoid arthritis, osteoarthritis, osteoporosis) |
| Hepatologic profile | The presence of chronic liver disease |
| Renal profile | The presence of chronic kidney disease |
| Oncologic profile | The presence of malignant neoplasms |
| Immunologic profile | The presence of human immunodeficiency virus infection and acquired immune deficiency syndrome |
| Multisystem multimorbidity | The coexistence of chronic conditions affecting two or more different organ systems |
| Complex multimorbidity | The coexistence of chronic conditions affecting three or more different organ systems |
| Polypharmacy | The concurrent supply of five or more medicines over a period of at least six months, allowing for a permissible gap of up to 30 calendar days between dispensations of the same medicine |
| Excessive polypharmacy | The concurrent supply of ten or more medicines over a period of at least six months, allowing for a permissible gap of up to 30 calendar days between dispensations of the same medicine |
| Anticholinergic activity | Supply of one or more medicines with anticholinergic activity |
| High anticholinergic score | Supply of one or more medicines resulting in a cumulative anticholinergic score of 3 or higher |
| High-risk medicine for chronic patients | Supply of at least one medicine classified as high-risk in the context of chronic disease management |

**Table S3: Medicines in ATC Group D (Dermatologicals) considered “chronic” in this study.**

| Medicines by ATC* group | | Duration |
| --- | --- | --- |
| **D05A** | **Antipsoriatics for topical use** |  |
| D05AA | Tars | 30 days |
| D05AD | Psoralens for topical use | 30 days |
| D05AX | Other antipsoriatics for topical use | 30 days |
| **D07A** | **Corticosteroids, plain** |  |
| D07AA | Corticosteroids, weak (group I) | 30 days |
| D07AB | Corticosteroids, moderately potent (group II) | 30 days |
| D07AC | Corticosteroids, potent (group III) | 30 days |
| D07AD | Corticosteroids, very potent (group IV) | 30 days |
| **D11A** | **Other dermatological preparations** |  |
| D11AX | Other dermatologicals | 30 days |
| **R01A** | **Decongestants and other nasal preparations for topical use** |  |
| R01AD | Corticosteroids | 30 days |
| **S01E** | **Antiglaucoma preparations and miotics** |  |
| S01EA | Sympathomimetics in glaucoma therapy | Chronic use by default |
| S01EB | Parasympathomimetics | Chronic use by default |
| S01EC | Carbonic anhydrase inhibitors | Chronic use by default |
| S01ED | Beta blocking agents | Chronic use by default |
| S01EE | Prostaglandin analogues | Chronic use by default |
| S01EX | Other antiglaucoma preparations | Chronic use by default |

***** ATC: Anatomical Therapeutic Chemical classification system *(WHO Collaborating Centre for Drug Statistics Methodology, ATC classification index with DDDs 2024 Oslo, Norway 2024)*

**Table S4: High-risk medicines for chronic patients.**

| High-risk medicines for chronic patients, by ATC^‡^ code | |
| --- | --- |
| A10A | Insulins and analogues |
| A10B | Blood glucose lowering drugs, excl. insulins |
| B01AA | Vitamin K antagonists |
| B01AC | Platelet aggregation inhibitors excl. heparin |
| B01AE | Direct thrombin inhibitors |
| B01AF | Direct factor Xa inhibitors |
| B01AX | Other antithrombotic agents |
| C01AA05 | Digoxin |
| C01BD01 | Amiodarone |
| C01BD07 | Dronedarone |
| C03C | High-ceiling diuretics |
| C03DA01 | Spironolactone |
| C03DA04 | Eplerenone |
| C07A | Beta blocking agents |
| H02A | Corticosteroids for systemic use, plain |
| L01 | Antineoplastic agents |
| L04A | Immunosuppressants |
| L04AX03 | Methotrexate† |
| M01A | Antiinflammatory and antirheumatic products, non-steroids |
| N02A | Opioids |
| N02BA01 | Acetylsalicylic acid |
| N03AB02 | Phenytoin |
| N03AF01 | Carbamazepine |
| N03AG01 | Valproic acid |
| N05A | Antipsychotics |
| N05B | Anxiolytics |
| N05C | Hypnotics and sedatives |

High-risk medicines for chronic patients (MARC, by its initials in Spanish), according to the list published by the Ministry of Health *(Proyecto MARC: Elaboración de una Lista de Medicamentos de Alto Riesgo para los Pacientes Crónicos. Informe 2014. Ministerio de Sanidad, Servicios Sociales e Igualdad, España).*

**^‡^** ATC: Anatomical Therapeutic Chemical classification system *(WHO Collaborating Centre for Drug Statistics Methodology, ATC classification index with DDDs 2024 Oslo, Norway 2024).*

**^†^** Non-oncological use.

**Table S5: Anticholinergic activity scores.**

| ATC^‡^ | SCORE* | ATC | SCORE | ATC | SCORE | ATC | SCORE | ATC | SCORE | ATC | SCORE |
| --- | --- | --- | --- | --- | --- | --- | --- | --- | --- | --- | --- |
| A01AC01 | 1 | C05AA01 | 1 | G04BD02 | 3 | N02AJ17 | 1 | N05CM05 | 3 | R06AD52 | 3 |
| A01AC02 | 1 | C05AA04 | 1 | G04BD04 | 3 | N02AJ18 | 1 | N06AA01 | 3 | R06AE05 | 3 |
| A01AC03 | 1 | C05AA09 | 1 | G04BD07 | 3 | N02AJ19 | 1 | N06AA02 | 3 | R06AE55 | 3 |
| A01AC54 | 1 | C05AA12 | 1 | G04BD10 | 3 | N02AX02 | 1 | N06AA03 | 3 | R06AX02 | 2 |
| A02BA01 | 2 | C05AE02 | 1 | H02AB02 | 1 | N03AE01 | 1 | N06AA04 | 3 | S01AA11 | 1 |
| A02BA02 | 2 | C08DB01 | 1 | H02AB04 | 1 | N03AF01 | 2 | N06AA06 | 3 | S01AA19 | 1 |
| A02BA03 | 1 | C09AA01 | 1 | H02AB06 | 1 | N03AF02 | 2 | N06AA09 | 3 | S01BA01 | 1 |
| A02BA04 | 1 | C09BA01 | 1 | H02AB07 | 1 | N03AG01 | 1 | N06AA10 | 3 | S01BA02 | 1 |
| A02BA07 | 2 | D04AA02 | 3 | H02AB08 | 1 | N04AA01 | 3 | N06AA11 | 3 | S01BA03 | 1 |
| A02BA51 | 2 | D04AA10 | 3 | H02AB09 | 1 | N04AA04 | 3 | N06AA12 | 3 | S01BA04 | 1 |
| A02BA53 | 1 | D04AA32 | 3 | H02AB10 | 1 | N04AB02 | 3 | N06AB03 | 1 | S01BA05 | 1 |
| A03AA07 | 3 | D06AX07 | 1 | H02BX01 | 1 | N04AC01 | 3 | N06AB05 | 2 | S01BB01 | 1 |
| A03AB05 | 3 | D07AA01 | 1 | J01CA01 | 1 | N04BB01 | 1 | N06AB06 | 1 | S01BB02 | 1 |
| A03BA01 | 3 | D07AA02 | 1 | J01CA12 | 1 | N04BC01 | 1 | N06AB08 | 1 | S01CA01 | 1 |
| A03BA03 | 3 | D07AA03 | 1 | J01CA51 | 1 | N05AA01 | 3 | N06AF03 | 1 | S01CA02 | 1 |
| A03CA34 | 3 | D07AB02 | 1 | J01CR01 | 1 | N05AA02 | 2 | N06CA01 | 3 | S01CA03 | 1 |
| A03CB03 | 3 | D07AB09 | 1 | J01CR05 | 1 | N05AB02 | 1 | N06CA03 | 1 | S01CA08 | 1 |
| A03CB31 | 3 | D07AB11 | 1 | J01DB03 | 1 | N05AB03 | 1 | R01AD02 | 1 | S01CB01 | 1 |
| A04AD01 | 3 | D07AB19 | 1 | J01DC01 | 1 | N05AB04 | 1 | R01AD03 | 1 | S01CB02 | 1 |
| A04AD51 | 3 | D07AC14 | 1 | J01DC03 | 1 | N05AB06 | 2 | R01AD11 | 1 | S01CB03 | 1 |
| A07AA09 | 1 | D07AC16 | 1 | J01FF01 | 1 | N05AC02 | 3 | R01AD52 | 1 | S01FA01 | 3 |
| A07DA03 | 1 | D07BA01 | 1 | J01GB03 | 1 | N05AE02 | 2 | R01AD53 | 1 | S01FA02 | 3 |
| A07DA05 | 1 | D07BA04 | 1 | J01XA01 | 1 | N05AF04 | 1 | R01AD60 | 1 | S01GX08 | 1 |
| A07DA52 | 1 | D07BB03 | 1 | J04AB01 | 1 | N05AG02 | 2 | R03AK06 | 1 | S01XA18 | 1 |
| A07DA53 | 1 | D07BB04 | 1 | L04AD01 | 1 | N05AH02 | 3 | R03BA06 | 1 | S02AA14 | 1 |
| A07EA01 | 1 | D07CA01 | 1 | L04AX01 | 1 | N05AH03 | 3 | R03DA04 | 1 | S02BA01 | 1 |
| A07EA02 | 1 | D07CA02 | 1 | M03AC01 | 1 | N05BA01 | 1 | R03DA12 | 3 | S02BA03 | 1 |
| A07EA03 | 1 | D07CA03 | 1 | M03BC01 | 3 | N05BA02 | 1 | R03DA54 | 1 | S02BA06 | 1 |
| B01AC07 | 1 | D07CB01 | 1 | M03BC51 | 3 | N05BA04 | 1 | R03DA74 | 1 | S02CA01 | 1 |
| C01AA04 | 1 | D07CB04 | 1 | M03BX08 | 2 | N05BA05 | 1 | R03DB04 | 1 | S02CA03 | 1 |
| C01BA03 | 2 | D07XA01 | 1 | N02AA05 | 1 | N05BA06 | 1 | R06AA02 | 3 | S02CA04 | 1 |
| C01DA08 | 1 | D07XA02 | 1 | N02AA55 | 1 | N05BA12 | 1 | R06AA04 | 3 | S02CA06 | 1 |
| C01DA14 | 1 | D07XB02 | 1 | N02AA56 | 1 | N05BA56 | 1 | R06AA08 | 3 | S03AA06 | 1 |
| C01DA58 | 1 | D07XB05 | 1 | N02AB02 | 2 | N05BB01 | 3 | R06AA52 | 3 | S03BA01 | 1 |
| C02DB02 | 1 | D10AA02 | 1 | N02AB52 | 2 | N05BB51 | 3 | R06AA54 | 3 | S03BA02 | 1 |
| C02LG02 | 1 | D10AA03 | 1 | N02AB72 | 2 | N05CD01 | 1 | R06AB01 | 3 | S03CA01 | 1 |
| C03BA04 | 1 | D10AF01 | 1 | N02AG03 | 2 | N05CD04 | 1 | R06AB04 | 3 | S03CA02 | 1 |
| C03BB04 | 1 | D10AF51 | 1 | N02AJ13 | 1 | N05CD05 | 1 | R06AB51 | 3 | S03CA04 | 1 |
| C03DB02 | 1 | G01AA10 | 1 | N02AJ14 | 1 | N05CD07 | 1 | R06AC01 | 3 | V03AB05 | 3 |
| C03EA06 | 1 | G02CB01 | 1 | N02AJ15 | 1 | N05CD08 | 1 | R06AD02 | 3 | N05AH04 | 2 |

*****Based on the Anticholinergic Drug Scale *(Carnahan, R. M., et al., 2006, Journal of clinical pharmacology, 46, 1481–1486)* and updated versions *(Eum, S., et al., 2017, Schizophrenia research, 190, 129–135).*

**^‡^** ATC: Anatomical Therapeutic Chemical classification system *(https://atcddd.fhi.no/).*

**Statistical analysis – technical details**

Logistic regression models were implemented in Python (version 3.12) using *scikit-learn* (Pedregosa et al, 2011). Class imbalance was addressed with the Synthetic Minority Over-sampling Technique (SMOTE) from the *imbalanced-learn* package (Lemaître et al, 2017). Gradient boosting models were implemented using XGBoost (Chen & Guestrin, 2016). Data preprocessing included handling missing values, one-hot encoding of categorical variables, and standardisation of continuous variables using *scikit-learn*. Feature importance was extracted from the fitted models to identify the most relevant predictors.

Hyperparameters were tuned using a random 5% sample of the dataset, employing stratified 5-fold cross-validation. Performance was evaluated with metrics calculated using *scikit-learn* (Pedregosa et al, 2011), *NumPy* (Harris et al, 2020), *Pandas* (McKinney, 2010), and visualisations were produced using *Matplotlib* (Hunter, 2007) and *Seaborn* (Waskom, 2021).

Age at onset thresholds for each outcome were calculated in R (version 4.4.2) using the *cutpointr* package (Thiele & Hirschfeld, 2021), with the Youden index applied to identify optimal cut-points.

All code was executed in a secure, high-performance computing environment without internet access.

**References**

Chen T, Guestrin C. XGBoost: A scalable tree boosting system. In: Proceedings of the 22nd ACM SIGKDD International Conference on Knowledge Discovery and Data Mining. New York, NY: ACM; 2016:785–94.

*“Cutpointr”* R software package, https://cran.r-project.org/web/packages/cutpointr/vignettes/cutpointr.html (accessed Aug 11, 2025).

Harris CR, Millman KJ, van der Walt SJ, et al. Array programming with NumPy. Nature. 2020;585(7825):357–62.

Hunter JD. Matplotlib: A 2D graphics environment. Comput Sci Eng. 2007;9(3):90–5.

Lemaître G, Nogueira F, Aridas CK. Imbalanced-learn: A Python Toolbox to Tackle the Curse of Imbalanced Datasets in Machine Learning. J Mach Learn Res. 2017;18(17):1–5.

McKinney W. Pandas: Python Data Analysis Library. Zenodo. 2010. Available from: https://pandas.pydata.org/2 (accessed Aug 11, 2025).

Pedregosa F, Varoquaux G, Gramfort A, et al. Scikit-learn: Machine learning in Python. J Mach Learn Res. 2011;12:2825–30.

Thiele C, Hirschfeld G. Cutpointr: Improved Estimation and Validation of Optimal Cutpoints in R. J Stat Softw. 2021;98(11):1–27.

Waskom ML. Seaborn: Statistical data visualization. J Open Source Softw. 2021;6(60):3021.

Youden WJ. Index for rating diagnostic tests. Cancer 1950; 3: 32–5.

**Table S6: Clinical characteristics of the study population by sex and age group.**

|  |  | **All ages** | **0-14 years** | **15-44 years** | **45-64 years** | **65-79 years** | **≥80 years** |
| --- | --- | --- | --- | --- | --- | --- | --- |
| **Total population** | |  |  |  |  |  |  |
|  | Population | 17 423 343 (100.00) | 2 526 000 (14.50) | 6 456 425 (37.06) | 5 108 451 (29.32) | 2 256 135 (12.95) | 1 076 332 (6.18) |
|  | Age, mean (SD^*^) | 42.88 (23.15) | 7.28 (4.38) | 31.01 (8.77) | 53.83 (5.68) | 71.37 (4.22) | 86.04 (4.66) |
|  | At least one chronic condition | 6 738 993 (38.68) | 301 058 (11.92) | 1 382 009 (21.41) | 2 330 109 (45.61) | 1 766 523 (78.30) | 959 294 (89.13) |
|  | Multimorbidity^†^ | 2 922 855 (16.78) | 16 140 (0.64) | 217 008 (3.36) | 845 171 (16.54) | 1 100 263 (48.77) | 744 273 (69.15) |
|  | Polypharmacy^‡^ | 948 346 (5.44) | 491 (0.02) | 20 908 (0.32) | 210 687 (4.12) | 402 004 (17.82) | 314 256 (29.20) |
|  | High-risk medicines^§^ | 2 227 561 (12.78) | 60 452 (2.39) | 177 757 (2.75) | 706 727 (13.83) | 781 009 (34.62) | 501 616 (46.60) |
| ***People with at least one chronic condition*** | |  |  |  |  |  |  |
|  | Multimorbidity | 2 922 855 (43.37) | 16 140 (5.36) | 217 008 (15.70) | 845 171 (36.27) | 1 100 263 (62.28) | 744 273 (77.59) |
|  | Multisystem multimorbidity^††^ | 2 438 114 (36.18) | 15 614 (5.19) | 186 883 (13.52) | 674 969 (28.97) | 905 709 (51.27) | 654 939 (68.27) |
|  | Complex multimorbidity^**^ | 765 211 (11.35) | 545 (0.18) | 17 424 (1.26) | 139 178 (5.97) | 308 165 (17.44) | 299 899 (31.26) |
|  | Polypharmacy | 851 776 (12.64) | 209 (0.07) | 13 615 (0.99) | 176 456 (7.57) | 367 135 (20.78) | 294 361 (30.69) |
|  | Excessive polypharmacy^‡‡^ | 107 287 (1.59) | <10 (0.00) | 1 032 (0.07) | 17 617 (0.76) | 46 242 (2.62) | 42 392 (4.42) |
|  | High-risk medicines | 1 795 320 (26.64) | 12 587 (4.18) | 94 538 (6.84) | 529 072 (22.71) | 692 229 (39.19) | 466 894 (48.70) |
|  | Anticholinergic activity medicines^▲^ | 830 471 (12.32) | 4 134 (1.37) | 64 944 (4.70) | 262 487 (11.27) | 291 077 (16.48) | 207 829 (21.67) |
|  | High anticholinergic burden^▲▲^ | 133 732 (1.98) | 251 (0.08) | 13 641 (0.99) | 49 913 (2.14) | 42 165 (2.39) | 27 762 (2.89) |
| **Women** | |  |  |  |  |  |  |
|  | Population | 8 934 222 (51.28) | 1 227 790 (13.74) | 3 232 962 (36.19) | 2 579 284 (28.87) | 1 214 172 (13.59) | 680 014 (7.61) |
|  | Age, mean (SD) | 44.09 (23.60) | 7.28 (4.38) | 31.08 (8.72) | 53.90 (5.69) | 71.47 (4.23) | 86.37 (4.79) |
|  | At least one chronic condition | 3 680 095 (41.19) | 130 368 (10.62) | 724 380 (22.41) | 1 237 019 (47.96) | 974 042 (80.22) | 614 286 (90.33) |
|  | Multimorbidity | 1 729 253 (19.36) | 6 984 (0.57) | 122 774 (3.80) | 472 942 (18.34) | 635 720 (52.36) | 490 833 (72.18) |
|  | Polypharmacy | 540 570 (6.05) | 205 (0.02) | 11 712 (0.36) | 108 690 (4.21) | 216 956 (17.87) | 203 007 (29.85) |
|  | High-risk medicines | 1 236 253 (13.84) | 28 335 (2.31) | 100 290 (3.10) | 368 052 (14.27) | 417 421 (34.38) | 322 155 (47.37) |
| ***Women with at least one chronic condition*** | |  |  |  |  |  |  |
|  | Multimorbidity | 1 729 253 (46.99) | 6 984 (5.36) | 122 774 (16.95) | 472 942 (38.23) | 635 720 (65.27) | 490 833 (79.90) |
|  | Multisystem multimorbidity | 1 527 602 (41.51) | 6 719 (5.15) | 105 695 (14.59) | 410 762 (33.21) | 560 270 (57.52) | 444 156 (72.30) |
|  | Complex multimorbidity | 530 359 (14.41) | 243 (0.19) | 10 596 (1.46) | 95 222 (7.70) | 209 841 (21.54) | 214 457 (34.91) |
|  | Polypharmacy | 485 946 (13.20) | 85 (0.07) | 7 522 (1.04) | 89 663 (7.25) | 198 208 (20.35) | 190 468 (31.01) |
|  | Excessive polypharmacy | 65 252 (1.77) | <10 (0.00) | 626 (0.09) | 9 450 (0.76) | 26 611 (2.73) | 28 563 (4.65) |
|  | High-risk medicines | 996 880 (27.09) | 5 495 (4.21) | 51 236 (7.07) | 267 942 (21.66) | 370 822 (38.07) | 301 385 (49.06) |
|  | Anticholinergic activity medicines | 554 018 (15.05) | 1 612 (1.24) | 38 504 (5.32) | 167 840 (13.57) | 195 308 (20.05) | 150 754 (24.54) |
|  | High anticholinergic burden | 90 426 (2.46) | 105 (0.08) | 6 926 (0.96) | 31 738 (2.57) | 30 503 (3.13) | 21 154 (3.44) |
| **Men** | |  |  |  |  |  |  |
|  | Population | 8 489 121 (48.72) | 1 298 210 (15.29) | 3 223 463 (37.97) | 2 529 167 (29.79) | 1 041 963 (12.27) | 396 318 (4.67) |
|  | Age, mean (SD) | 41.61 (22.59) | 7.28 (4.38) | 30.94 (8.83) | 53.75 (5.67) | 71.25 (4.20) | 85.48 (4.38) |
|  | At least one chronic condition | 3 058 898 (36.03) | 170 690 (13.15) | 657 629 (20.40) | 1 093 090 (43.22) | 792 481 (76.06) | 345 008 (87.05) |
|  | Multimorbidity | 1 193 602 (14.06) | 9 156 (0.71) | 94 234 (2.92) | 372 229 (14.72) | 464 543 (44.58) | 253 440 (63.95) |
|  | Polypharmacy | 407 776 (4.80) | 286 (0.02) | 9 196 (0.29) | 101 997 (4.03) | 185 148 (17.76) | 111 249 (28.07) |
|  | High-risk medicines | 991 308 (11.68) | 32117 (2.47) | 77 467 (2.40) | 338 675 (13.39) | 363 588 (34.89) | 179 461 (45.28) |
| ***Men with at least one chronic condition*** | |  |  |  |  |  |  |
|  | Multimorbidity | 1 193 602 (39.02) | 9 156 (5.36) | 94 234 (14.33) | 372 229 (34.05) | 464 543 (58.62) | 253 440 (73.46) |
|  | Multisystem multimorbidity | 910 512 (29.77) | 8 895 (5.21) | 81 188 (12.35) | 264 207 (24.17) | 345 439 (43.59) | 210 783 (61.10) |
|  | Complex multimorbidity | 234 852 (7.68) | 302 (0.18) | 6 828 (1.04) | 43 956 (4.02) | 98 324 (12.41) | 85 442 (24.77) |
|  | Polypharmacy | 365 830 (11.96) | 124 (0.07) | 6 093 (0.93) | 86 793 (7.94) | 168 927 (21.32) | 103 893 (30.11) |
|  | Excessive polypharmacy | 42 035 (1.37) | <10 (0.00) | 406 (0.06) | 8 167 (0.75) | 19 631 (2.48) | 13 829 (4.01) |
|  | High-risk medicines | 798 440 (26.10) | 7092 (4.15) | 43 302 (6.58) | 261 130 (23.89) | 321 407 (40.56) | 165 509 (47.97) |
|  | Anticholinergic activity medicines | 276 453 (9.04) | 2522 (1.48) | 26 440 (4.02) | 94 647 (8.66) | 95 769 (12.08) | 57 075 (16.54) |
|  | High anticholinergic burden | 43 306 (1.42) | 146 (0.09) | 6 715 (1.02) | 18 175 (1.66) | 11 662 (1.47) | 6 608 (1.92) |

Data are presented as number of people, with percentages in parentheses, unless otherwise specified. ^*^*SD* = standard deviation. ^†^Multimorbidity: ≥2 chronic conditions. ^††^Multisystem multimorbidity: Conditions affecting ≥2 organ systems. ^**^Complex multimorbidity: Conditions affecting ≥3 organ systems. ^‡^Polypharmacy: ≥5 concurrent medicines for ≥6 months (≤30-day gaps). ^‡‡^Excessive polypharmacy ≥10 concurrent medicines for ≥6 months (≤30-day gaps). ^§^High-risk medicine: ≥1 medicine classified as high-risk for chronic patients. ^▲^Anticholinergic activity: ≥1 medicine with known anticholinergic properties. ^▲▲^High anticholinergic burden: Anticholinergic score ≥3.

**Table S7: Prevalence of chronic conditions in the general population (overall and with comorbidity), stratified by sex and age group.**

|  | Hypertension | Diabetes mellitus | Malignant neoplasms | Ischaemic heart disease | COPD | Depression | Stroke | Asthma | Chronic kidney disease |
| --- | --- | --- | --- | --- | --- | --- | --- | --- | --- |
| **Overall** | |  |  |  |  |  |  |  |  |
| **All** | **3 194 597 (18.34)** | **1 149 157 (6.60)** | **762 990 (4.38)** | **416 133 (2.39)** | **283 822 (1.63)** | **1 061 958 (6.10)** | **152 614 (0.88)** | **1 238 355 (7.11)** | **219 122 (1.26)** |
| *0-14 years* | 4 036 (0.16) | 4 579 (0.18) | 31 614 (1.25) | 4 358 (0.17) | 1 045 (0.04) | 2 480 (0.10) | 1 330 (0.05) | 216 670 (8.58) | 241 (0.01) |
| *15-44 years* | 194 086 (3.01) | 62 309 (0.97) | 84 874 (1.31) | 24 442 (0.38) | 7 311 (0.11) | 201 543 (3.12) | 5 648 (0.09) | 545 535 (8.45) | 4 274 (0.07) |
| *45-64 years* | 1 094 663 (21.43) | 352 127 (6.89) | 225 836 (4.42) | 111 986 (2.19) | 80 520 (1.58) | 413 799 (8.10) | 31 813 (0.62) | 279 634 (5.47) | 26 466 (0.52) |
| *65-79 years* | 1 172 939 (51.99) | 464 926 (20.61) | 260 256 (11.54) | 159 847 (7.08) | 119 810 (5.31) | 277 624 (12.31) | 53 666 (2.38) | 128 154 (5.68) | 76 717 (3.40) |
| ≥*80 years* | 728 873 (67.72) | 265 216 (24.64) | 160 410 (14.9) | 115 500 (10.73) | 75 136 (6.98) | 166 512 (15.47) | 60 157 (5.59) | 68 362 (6.35) | 111 424 (10.35) |
| **Women** | **1 682 017 (18.83)** | **523 820 (5.86)** | **422 495 (4.73)** | **149 043 (1.67)** | **93 422 (1.05)** | **770 084 (8.62)** | **71 700 (0.80)** | **669 923 (7.50)** | **114 424 (1.28)** |
| *0-14 years* | 1 861 (0.15) | 2 186 (0.18) | 15 095 (1.23) | 2 000 (0.16) | 491 (0.04) | 1 330 (0.11) | 612 (0.05) | 88 036 (7.17) | 76 (0.01) |
| *15-44 years* | 79 628 (2.46) | 27 773 (0.86) | 51 544 (1.59) | 9 526 (0.29) | 3 031 (0.09) | 135 661 (4.20) | 2 701 (0.08) | 266 840 (8.25) | 1 569 (0.05) |
| *45-64 years* | 492 049 (19.08) | 129 843 (5.03) | 144 184 (5.59) | 30 491 (1.18) | 31 920 (1.24) | 290 772 (11.27) | 12 132 (0.47) | 173 470 (6.73) | 10 047 (0.39) |
| *65-79 years* | 629 478 (51.84) | 205 596 (16.93) | 128 338 (10.57) | 52 039 (4.29) | 34 907 (2.87) | 208 610 (17.18) | 21 699 (1.79) | 89 317 (7.36) | 33 961 (2.80) |
| ≥*80 years* | 479 001 (70.44) | 158 422 (23.3) | 83 334 (12.25) | 54 987 (8.09) | 23 073 (3.39) | 133 711 (19.66) | 34 556 (5.08) | 52 260 (7.69) | 68 771 (10.11) |
| **Men** | **1 512 580 (17.82)** | **625 337 (7.37)** | **340 495 (4.01)** | **267 090 (3.15)** | **190 400 (2.24)** | **291 874 (3.44)** | **80 914 (0.95)** | **568 432 (6.70)** | **104 698 (1.23)** |
| *0-14 years* | 2 175 (0.17) | 2 393 (0.18) | 16 519 (1.27) | 2 358 (0.18) | 554 (0.04) | 1 150 (0.09) | 718 (0.06) | 128 634 (9.91) | 165 (0.01) |
| *15-44 years* | 114 458 (3.55) | 34 536 (1.07) | 33 330 (1.03) | 14 916 (0.46) | 4 280 (0.13) | 65 882 (2.04) | 2 947 (0.09) | 278 695 (8.65) | 2 705 (0.08) |
| *45-64 years* | 602 614 (23.83) | 222 284 (8.79) | 81 652 (3.23) | 81 495 (3.22) | 48 600 (1.92) | 123 027 (4.86) | 19 681 (0.78) | 106 164 (4.20) | 16 419 (0.65) |
| *65-79 years* | 543 461 (52.16) | 259 330 (24.89) | 131 918 (12.66) | 107 808 (10.35) | 84 903 (8.15) | 69 014 (6.62) | 31 967 (3.07) | 38 837 (3.73) | 42 756 (4.10) |
| ≥*80 years* | 249 872 (63.05) | 106 794 (26.95) | 77 076 (19.45) | 60 513 (15.27) | 52 063 (13.14) | 32 801 (8.28) | 25 601 (6.46) | 16 102 (4.06) | 42 653 (10.76) |
| **With comorbidity** | | |  |  |  |  |  |  |  |
| **All** | **2 065 599 (11.86)** | **911 038 (5.23)** | **535 355 (3.07)** | **340 146 (1.95)** | **234 378 (1.35)** | **733 599 (4.21)** | **131 926 (0.76)** | **453 377 (2.60)** | **202 374 (1.16)** |
| *0-14 years* | 1 005 (0.04) | 872 (0.03) | 4 899 (0.19) | 1 108 (0.04) | 384 (0.02) | 661 (0.03) | 348 (0.01) | 13715 (0.54) | 66 (0.00) |
| *15-44 years* | 61 368 (0.95) | 23 208 (0.36) | 28 407 (0.44) | 9 588 (0.15) | 3 567 (0.06) | 75 970 (1.18) | 2 323 (0.04) | 108 906 (1.69) | 2 211 (0.03) |
| *45-64 years* | 539 302 (10.56) | 240 320 (4.70) | 132 695 (2.60) | 78 246 (1.53) | 56 660 (1.11) | 250 430 (4.90) | 23 449 (0.46) | 152 189 (2.98) | 21 181 (0.41) |
| *65-79 years* | 848 708 (37.62) | 399 203 (17.69) | 219 508 (9.73) | 141 355 (6.27) | 103 598 (4.59) | 246 007 (10.90) | 48 613 (2.15) | 112 853 (5.00) | 71 279 (3.16) |
| *≥80 years* | 615 216 (57.16) | 247 435 (22.99) | 149 846 (13.92) | 109 849 (10.21) | 70 169 (6.52) | 160 531 (14.91) | 57 193 (5.31) | 65 714 (6.11) | 107 637 (10.00) |
| **Women** | **1 190 031 (13.32)** | **436 971 (4.89)** | **293 373 (3.28)** | **129 346 (1.45)** | **78 503 (0.88)** | **540 864 (6.05)** | **63 419 (0.71)** | **297 752 (3.33)** | **107 359 (1.2)** |
| *0-14 years* | 406 (0.03) | 392 (0.03) | 2 068 (0.17) | 458 (0.04) | 197 (0.02) | 333 (0.03) | 163 (0.01) | 5 754 (0.47) | 21 (0.00) |
| *15-44 years* | 28 747 (0.89) | 10 737 (0.33) | 17 300 (0.54) | 3 824 (0.12) | 1 585 (0.05) | 49 765 (1.54) | 1 130 (0.03) | 60 702 (1.88) | 834 (0.03) |
| *45-64 years* | 269 896 (10.46) | 93 566 (3.63) | 83 936 (3.25) | 23 199 (0.90) | 23 504 (0.91) | 175 442 (6.80) | 8955 (0.35) | 100 569 (3.90) | 8 006 (0.31) |
| *65-79 years* | 478 596 (39.42) | 182 680 (15.05) | 110 577 (9.11) | 48 383 (3.98) | 31 038 (2.56) | 185 798 (15.30) | 20 010 (1.65) | 80 152 (6.60) | 31 761 (2.62) |
| *≥80 years* | 412 386 (60.64) | 149 596 (22.00) | 79 492 (11.69) | 53 482 (7.86) | 22 179 (3.26) | 129 526 (19.05) | 33 161 (4.88) | 50 575 (7.44) | 66 737 (9.81) |
| **Men** | **875 568 (10.31)** | **474 067 (5.58)** | **241 982 (2.85)** | **210 800 (2.48)** | **155 875 (1.84)** | **192 735 (2.27)** | **68 507 (0.81)** | **155 625 (1.83)** | **95 015 (1.12)** |
| *0-14 years* | 599 (0.05) | 480 (0.04) | 2 831 (0.22) | 650 (0.05) | 187 (0.01) | 328 (0.03) | 185 (0.01) | 7 961 (0.61) | 45 (0.00) |
| *15-44 years* | 32 621 (1.01) | 12 471 (0.39) | 11 107 (0.34) | 5 764 (0.18) | 1 982 (0.06) | 26 205 (0.81) | 1 193 (0.04) | 48 204 (1.50) | 1 377 (0.04) |
| *45-64 years* | 269 406 (10.65) | 146 754 (5.80) | 48 759 (1.93) | 55 047 (2.18) | 33 156 (1.31) | 74 988 (2.96) | 14 494 (0.57) | 51 620 (2.04) | 13 175 (0.52) |
| *65-79 years* | 370 112 (35.52) | 216 523 (20.78) | 108 931 (10.45) | 92 972 (8.92) | 72 560 (6.96) | 60 209 (5.78) | 28 603 (2.75) | 32 701 (3.14) | 39 518 (3.79) |
| *≥80 years* | 202 830 (51.18) | 97 839 (24.69) | 70 354 (17.75) | 56 367 (14.22) | 47 990 (12.11) | 31 005 (7.82) | 24 032 (6.06) | 15 139 (3.82) | 40 900 (10.32) |

(Table S7 continues on next page)

**Table S7: Prevalence of chronic conditions in the general population (overall and with comorbidity), stratified by sex and age group.**

(continued from previous page)

|  | Dementia | Mental health disorders | Osteoporosis | Rheumatoid arthritis | Liver disease | HIV/AIDS | Osteoarthritis | Heart failure | Parkinson's disease |
| --- | --- | --- | --- | --- | --- | --- | --- | --- | --- |
| **Overall** | |  |  |  |  |  |  |  |  |
| **All** | **98 569 (0.57)** | **899 986 (5.17)** | **464 950 (2.67)** | **90 663 (0.52)** | **21 498 (0.12)** | **39 630 (0.23)** | **1 433 647 (8.23)** | **166 718 (0.96)** | **49 472 (0.28)** |
| *0-14 years* | 222 (0.01) | 40 956 (1.62) | 88 (0.00) | 1 050 (0.04) | 68 (0.00) | 298 (0.01) | 8 222 (0.33) | 417 (0.02) | 244 (0.01) |
| *15-44 years* | 448 (0.01) | 322 685 (5.00) | 2 666 (0.04) | 10 463 (0.16) | 1 066 (0.02) | 13 920 (0.22) | 145 806 (2.26) | 3 844 (0.06) | 885 (0.01) |
| *45-64 years* | 3 157 (0.06) | 343 662 (6.73) | 82 615 (1.62) | 32 206 (0.63) | 9 651 (0.19) | 22 857 (0.45) | 378 373 (7.41) | 17 320 (0.34) | 5 133 (0.10) |
| *65-79 years* | 21 852 (0.97) | 131 665 (5.84) | 221 515 (9.82) | 29 144 (1.29) | 7 883 (0.35) | 2334 (0.10) | 515 030 (22.83) | 46 729 (2.07) | 18 652 (0.83) |
| ≥*80 years* | 72 890 (6.77) | 61 018 (5.67) | 158 066 (14.69) | 17 800 (1.65) | 2 830 (0.26) | 221 (0.02) | 386 216 (35.88) | 98 408 (9.14) | 24 558 (2.28) |
| **Women** | **69 858 (0.78)** | **574 356 (6.43)** | **441 598 (4.94)** | **66 676 (0.75)** | **9 288 (0.1)** | **8 865 (0.1)** | **984 723 (11.02)** | **92 722 (1.04)** | **25 611 (0.29)** |
| *0-14 years* | 102 (0.01) | 20 948 (1.71) | 43 (0.00) | 673 (0.05) | 32 (0.00) | 140 (0.01) | 3 734 (0.30) | 189 (0.02) | 127 (0.01) |
| *15-44 years* | 218 (0.01) | 195 218 (6.04) | 1 848 (0.06) | 7 636 (0.24) | 534 (0.02) | 2 543 (0.08) | 78 987 (2.44) | 1 298 (0.04) | 445 (0.01) |
| *45-64 years* | 1 509 (0.06) | 218 316 (8.46) | 77 637 (3.01) | 23 823 (0.92) | 3 632 (0.14) | 5 638 (0.22) | 258 286 (10.01) | 5 938 (0.23) | 2 122 (0.08) |
| *65-79 years* | 13 565 (1.12) | 93 182 (7.67) | 212 365 (17.49) | 21 158 (1.74) | 3 423 (0.28) | 481 (0.04) | 359 163 (29.58) | 21 744 (1.79) | 8 692 (0.72) |
| ≥*80 years* | 54 464 (8.01) | 46 692 (6.87) | 149 705 (22.01) | 13 386 (1.97) | 1 667 (0.25) | 63 (0.01) | 284 553 (41.85) | 63 553 (9.35) | 14 225 (2.09) |
| **Men** | **28 711 (0.34)** | **325 630 (3.84)** | **23 352 (0.28)** | **23 987 (0.28)** | **12 210 (0.14)** | **30 765 (0.36)** | **448 924 (5.29)** | **73 996 (0.87)** | **23 861 (0.28)** |
| *0-14 years* | 120 (0.01) | 20 008 (1.54) | 45 (0.00) | 377 (0.03) | 36 (0.00) | 158 (0.01) | 4 488 (0.35) | 228 (0.02) | 117 (0.01) |
| *15-44 years* | 230 (0.01) | 127 467 (3.95) | 818 (0.03) | 2 827 (0.09) | 532 (0.02) | 11 377 (0.35) | 66 819 (2.07) | 2 546 (0.08) | 440 (0.01) |
| *45-64 years* | 1 648 (0.07) | 125 346 (4.96) | 4 978 (0.20) | 8 383 (0.33) | 6 019 (0.24) | 17 219 (0.68) | 120 087 (4.75) | 11 382 (0.45) | 3 011 (0.12) |
| *65-79 years* | 8 287 (0.8) | 38 483 (3.69) | 9 150 (0.88) | 7 986 (0.77) | 4 460 (0.43) | 1 853 (0.18) | 155 867 (14.96) | 24 985 (2.40) | 9 960 (0.96) |
| ≥*80 years* | 18 426 (4.65) | 14 326 (3.61) | 8 361 (2.11) | 4 414 (1.11) | 1 163 (0.29) | 158 (0.04) | 101 663 (25.65) | 34 855 (8.79) | 10333 (2.61) |
| **With comorbidity** | | |  |  |  |  |  |  |  |
| **All** | **87 949 (0.50)** | **462 292 (2.65)** | **394 312 (2.26)** | **66 081 (0.38)** | **17 026 (0.10)** | **19 658 (0.11)** | **1 082 793 (6.21)** | **154 357 (0.89)** | **43 075 (0.25)** |
| *0-14 years* | 109 (0.00) | 7 189 (0.28) | 34 (0.00) | 227 (0.01) | 27 (0.00) | 57 (0.00) | 2 117 (0.08) | 154 (0.01) | 66 (0.00) |
| *15-44 years* | 235 (0.00) | 94 813 (1.47) | 1 305 (0.02) | 3 568 (0.06) | 401 (0.01) | 3 752 (0.06) | 49 280 (0.76) | 1 997 (0.03) | 357 (0.01) |
| *45-64 years* | 2 245 (0.04) | 189 319 (3.71) | 55 051 (1.08) | 19 951 (0.39) | 6 899 (0.14) | 13 735 (0.27) | 230 298 (4.51) | 14 144 (0.28) | 3 367 (0.07) |
| *65-79 years* | 18 950 (0.84) | 113 117 (5.01) | 187 255 (8.30) | 25 441 (1.13) | 6 993 (0.31) | 1 912 (0.08) | 438 041 (19.42) | 43 826 (1.94) | 16 207 (0.72) |
| *≥80 years* | 66 410 (6.17) | 57 854 (5.38) | 150 667 (14.00) | 16 894 (1.57) | 2 706 (0.25) | 202 (0.02) | 363 057 (33.73) | 94 236 (8.76) | 23 078 (2.14) |
| **Women** | **63 078 (0.71)** | **308 391 (3.45)** | **374 368 (4.19)** | **49 221 (0.55)** | **7 395 (0.08)** | **5 160 (0.06)** | **767 185 (8.59)** | **87 286 (0.98)** | **23 083 (0.26)** |
| *0-14 years* | 44 (0.00) | 3 275 (0.27) | 20 (0.00) | 135 (0.01) | 14 (0.00) | 25 (0.00) | 917 (0.07) | 61 (0.00) | 29 (0.00) |
| *15-44 years* | 117 (0.00) | 57 761 (1.79) | 927 (0.03) | 2 674 (0.08) | 197 (0.01) | 855 (0.03) | 28 763 (0.89) | 743 (0.02) | 191 (0.01) |
| *45-64 years* | 1 084 (0.04) | 121 825 (4.72) | 51 512 (2.00) | 14 949 (0.58) | 2 509 (0.10) | 3 791 (0.15) | 159 809 (6.20) | 4 909 (0.19) | 1 483 (0.06) |
| *65-79 years* | 11 916 (0.98) | 81 001 (6.67) | 179 170 (14.76) | 18 676 (1.54) | 3 058 (0.25) | 428 (0.04) | 308 040 (25.37) | 20 505 (1.69) | 7 825 (0.64) |
| *≥80 years* | 49 917 (7.34) | 44 529 (6.55) | 142 739 (20.99) | 12 787 (1.88) | 1 617 (0.24) | 61 (0.01) | 269 656 (39.65) | 61 068 (8.98) | 13 555 (1.99) |
| **Men** | **24 871 (0.29)** | **153 901 (1.81)** | **19 944 (0.23)** | **16 860 (0.20)** | **9 631 (0.11)** | **14 498 (0.17)** | **315 608 (3.72)** | **67 071 (0.79)** | **19 992 (0.24)** |
| *0-14 years* | 65 (0.01) | 3 914 (0.30) | 14 (0.00) | 92 (0.01) | 13 (0.00) | 32 (0.00) | 1 200 (0.09) | 93 (0.01) | 37 (0.00) |
| *15-44 years* | 118 (0.00) | 37 052 (1.15) | 378 (0.01) | 894 (0.03) | 204 (0.01) | 2 897 (0.09) | 20 517 (0.64) | 1 254 (0.04) | 166 (0.01) |
| *45-64 years* | 1 161 (0.05) | 67 494 (2.67) | 3 539 (0.14) | 5 002 (0.20) | 4 390 (0.17) | 9 944 (0.39) | 70 489 (2.79) | 9 235 (0.37) | 1 884 (0.07) |
| *65-79 years* | 7 034 (0.68) | 32 116 (3.08) | 8 085 (0.78) | 6 765 (0.65) | 3 935 (0.38) | 1 484 (0.14) | 130 001 (12.48) | 23 321 (2.24) | 8 382 (0.80) |
| *≥80 years* | 16 493 (4.16) | 13 325 (3.36) | 7 928 (2.00) | 4 107 (1.04) | 1 089 (0.27) | 141 (0.04) | 93 401 (23.57) | 33 168 (8.37) | 9 523 (2.40) |

**Table S8: Thirty most frequently used high-risk medicines for chronic patients, stratified by sex and age group.**

| WOMEN |  |  |  |  |  |  |  |  |  |  |  |
| --- | --- | --- | --- | --- | --- | --- | --- | --- | --- | --- | --- |
| **0-14 years old** | | | | **15-44 years old** | | | | **45-64 years old** | | | |
|  |  | *n* | % |  |  | *n* | % |  |  | *n* | % |
| M01AE01 | ibuprofen | 24 531 | 2.00 | N05BA06 | lorazepam | 16 617 | 0.51 | N05BA06 | lorazepam | 66 649 | 2.58 |
| N03AG01 | valproic acid | 621 | 0.05 | M01AE01 | ibuprofen | 11 215 | 0.35 | B01AC06 | acetylsalicylic acid | 31 922 | 1.24 |
| H02AB06 | prednisolone | 620 | 0.05 | N05BA12 | alprazolam | 7 614 | 0.24 | N05BA12 | alprazolam | 29 856 | 1.16 |
| N05AX08 | risperidone | 401 | 0.03 | N05BA01 | diazepam | 5 931 | 0.18 | C07AB07 | bisoprolol | 28 863 | 1.12 |
| L04AX03 | methotrexate | 338 | 0.03 | M01AE02 | naproxen | 5 850 | 0.18 | N05CD06 | lormetazepam | 27 275 | 1.06 |
| A10AB04 | insulin lispro | 287 | 0.02 | M01AE17 | dexketoprofen | 5 466 | 0.17 | A10BA02 | metformin | 26 443 | 1.03 |
| A10AE04 | insulin glargine | 278 | 0.02 | N05CD06 | lormetazepam | 5 130 | 0.16 | N05BA01 | diazepam | 19 089 | 0.74 |
| A10AB05 | insulin aspart | 262 | 0.02 | B01AC06 | acetylsalicylic acid | 4 094 | 0.13 | N05BA08 | bromazepam | 18 609 | 0.72 |
| A10AE05 | insulin detemir | 128 | 0.01 | N05BA08 | bromazepam | 3 673 | 0.11 | N02AJ13 | tramadol and paracetamol | 18 520 | 0.72 |
| N05BA01 | diazepam | 125 | 0.01 | A10BA02 | metformin | 3 137 | 0.10 | N05CF02 | zolpidem | 17 275 | 0.67 |
| N05BA09 | clobazam | 123 | 0.01 | L04AX03 | methotrexate | 3 134 | 0.10 | M01AE01 | ibuprofen | 16 997 | 0.66 |
| N05BB01 | hydroxyzine | 123 | 0.01 | N05CF02 | zolpidem | 3 099 | 0.10 | L04AX03 | methotrexate | 12 784 | 0.50 |
| M01AE02 | naproxen | 104 | 0.01 | N05AH04 | quetiapine | 2 857 | 0.09 | M01AX25 | chondroitin sulfate | 11 804 | 0.46 |
| N05AX12 | aripiprazole | 98 | 0.01 | C07AB07 | bisoprolol | 2 767 | 0.09 | M01AE02 | naproxen | 8 800 | 0.34 |
| B01AC06 | acetylsalicylic acid | 85 | 0.01 | N02AJ13 | tramadol and paracetamol | 2 451 | 0.08 | N05AH04 | quetiapine | 8 703 | 0.34 |
| C07AA05 | propranolol | 83 | 0.01 | A10AB05 | insulin aspart | 2 395 | 0.07 | M01AE17 | dexketoprofen | 8 618 | 0.33 |
| L04AX01 | azathioprine | 80 | 0.01 | N05AX12 | aripiprazole | 2 238 | 0.07 | A10BD07 | metformin and sitagliptin | 7 577 | 0.29 |
| A10AE06 | insulin degludec | 70 | 0.01 | N03AG01 | valproic acid | 2 195 | 0.07 | C03CA01 | furosemide | 7 384 | 0.29 |
| M01AE17 | dexketoprofen | 66 | 0.01 | A10AE04 | insulin glargine | 1 800 | 0.06 | N02AX02 | tramadol | 6 523 | 0.25 |
| H02AB07 | prednisone | 63 | 0.01 | N05AH03 | olanzapine | 1 769 | 0.05 | N05BA05 | potassium clorazepate | 6 483 | 0.25 |
| H02AB09 | hydrocortisone | 60 | 0.00 | H02AB07 | prednisone | 1 634 | 0.05 | C07AB03 | atenolol | 6 479 | 0.25 |
| N05BA06 | lorazepam | 60 | 0.00 | L04AX01 | azathioprine | 1 595 | 0.05 | A10AE04 | insulin glargine | 6 441 | 0.25 |
| A10BA02 | metformin | 59 | 0.00 | C07AA05 | propranolol | 1 590 | 0.05 | M01AH05 | etoricoxib | 6 095 | 0.24 |
| L04AD02 | tacrolimus | 57 | 0.00 | N05BA05 | potassium clorazepate | 1 366 | 0.04 | C03CA04 | torasemide | 5 889 | 0.23 |
| A10AB06 | insulin glulisine | 52 | 0.00 | N05AX08 | risperidone | 1 254 | 0.04 | C07AA05 | propranolol | 5 636 | 0.22 |
| N05AH03 | olanzapine | 40 | 0.00 | A10AB04 | insulin lispro | 1 202 | 0.04 | H02AB07 | prednisone | 5 074 | 0.20 |
| N03AF01 | carbamazepine | 39 | 0.00 | A10AE06 | insulin degludec | 1 171 | 0.04 | B01AA07 | acenocoumarol | 4 984 | 0.19 |
| C07AB03 | atenolol | 31 | 0.00 | N02AX02 | tramadol | 1 034 | 0.03 | N05AH03 | olanzapine | 4 831 | 0.19 |
| H02AB04 | methylprednisolone | 30 | 0.00 | N03AF01 | carbamazepine | 899 | 0.03 | N03AG01 | valproic acid | 4 572 | 0.18 |
| N05CD08 | midazolam | 27 | 0.00 | M01AH05 | etoricoxib | 884 | 0.03 | A10BK01 | dapagliflozin | 4 472 | 0.17 |

(Table S8 continues on next page)

**Table S8: Thirty most frequently used high-risk medicines for chronic patients, stratified by sex and age group.**

(continued from previous page)

| WOMEN |  |  |  |  |  |  |  |
| --- | --- | --- | --- | --- | --- | --- | --- |
| **65-79 years old** | | | | **80 years and older** | | | |
|  |  | *n* | % |  |  | *n* | % |
| B01AC06 | acetylsalicylic acid | 76 099 | 6.27 | B01AC06 | acetylsalicylic acid | 76 756 | 11.29 |
| N05BA06 | lorazepam | 70 183 | 5.78 | C03CA01 | furosemide | 59 746 | 8.79 |
| C07AB07 | bisoprolol | 55 166 | 4.54 | N05BA06 | lorazepam | 51 556 | 7.58 |
| A10BA02 | metformin | 42 663 | 3.51 | C07AB07 | bisoprolol | 48 756 | 7.17 |
| N05CD06 | lormetazepam | 29 630 | 2.44 | A10BA02 | metformin | 24 854 | 3.65 |
| N02AJ13 | tramadol and paracetamol | 26 947 | 2.22 | N05CD06 | lormetazepam | 21 999 | 3.24 |
| C03CA01 | furosemide | 25 062 | 2.06 | N02AJ13 | tramadol and paracetamol | 21 791 | 3.20 |
| N05BA12 | alprazolam | 24 512 | 2.02 | B01AA07 | acenocoumarol | 20 088 | 2.95 |
| N05BA08 | bromazepam | 23 243 | 1.91 | C03CA04 | torasemide | 18 549 | 2.73 |
| N05CF02 | zolpidem | 20 274 | 1.67 | N05AH04 | quetiapine | 15 186 | 2.23 |
| M01AX25 | chondroitin sulfate | 15 414 | 1.27 | N05BA08 | bromazepam | 14 719 | 2.16 |
| C03CA04 | torasemide | 15 301 | 1.26 | N05BA12 | alprazolam | 14 634 | 2.15 |
| B01AA07 | acenocoumarol | 14 504 | 1.19 | B01AF02 | apixaban | 14 331 | 2.11 |
| A10BD07 | metformin and sitagliptin | 14 238 | 1.17 | N05CF02 | zolpidem | 12 413 | 1.83 |
| A10AE04 | insulin glargine | 12 291 | 1.01 | A10AE04 | insulin glargine | 10 060 | 1.48 |
| N05BA01 | diazepam | 12 264 | 1.01 | C03DA01 | spironolactone | 10 008 | 1.47 |
| C07AB03 | atenolol | 12 116 | 1.00 | B01AC04 | clopidogrel | 9 952 | 1.46 |
| L04AX03 | methotrexate | 9 512 | 0.78 | C07AG02 | carvedilol | 8 622 | 1.27 |
| A10BD08 | metformin and vildagliptin | 9 477 | 0.78 | B01AF01 | rivaroxaban | 8 439 | 1.24 |
| C07AG02 | carvedilol | 9 167 | 0.76 | A10BH05 | linagliptin | 7 812 | 1.15 |
| A10BX02 | repaglinide | 8 785 | 0.72 | A10BX02 | repaglinide | 7 727 | 1.14 |
| A10BH01 | sitagliptin | 8 388 | 0.69 | N02AB03 | fentanyl | 7 553 | 1.11 |
| B01AF02 | apixaban | 7 892 | 0.65 | C07AB03 | atenolol | 7 550 | 1.11 |
| M01AE01 | ibuprofen | 7 798 | 0.64 | C01AA05 | digoxin | 7 036 | 1.03 |
| N05AH04 | quetiapine | 7 709 | 0.63 | A10BD07 | metformin and sitagliptin | 6 817 | 1.00 |
| B01AC04 | clopidogrel | 7 192 | 0.59 | A10BH01 | sitagliptin | 6 646 | 0.98 |
| N02AX02 | tramadol | 7 174 | 0.59 | B01AF03 | edoxaban | 5 902 | 0.87 |
| C03DA01 | spironolactone | 6 621 | 0.55 | A10BD08 | metformin and vildagliptin | 5 397 | 0.79 |
| A10BK01 | dapagliflozin | 6 245 | 0.51 | N02AX02 | tramadol | 5 375 | 0.79 |
| C07AB12 | nebivolol | 6 241 | 0.51 | N05BA01 | diazepam | 5 235 | 0.77 |

(Table S8 continues on next page)

**Table S8: Thirty most frequently used high-risk medicines for chronic patients, stratified by sex and age group.**

(continued from previous page)

| MEN |  |  |  |  |  |  |  |  |  |  |  |
| --- | --- | --- | --- | --- | --- | --- | --- | --- | --- | --- | --- |
| **0-14 years old** | | | | **15-44 years old** | | | | **45-64 years old** | | | |
|  |  | *n* | % |  |  | *n* | % |  |  | *n* | % |
| M01AE01 | ibuprofen | 26 176 | 2.02 | N05BA06 | lorazepam | 8 732 | 0.27 | B01AC06 | acetylsalicylic acid | 69 431 | 2.75 |
| N05AX08 | risperidone | 1 736 | 0.13 | N05BA12 | alprazolam | 4 886 | 0.15 | C07AB07 | bisoprolol | 46 286 | 1.83 |
| N03AG01 | valproic acid | 1 004 | 0.08 | N03AG01 | valproic acid | 4 768 | 0.15 | A10BA02 | metformin | 38 416 | 1.52 |
| H02AB06 | prednisolone | 882 | 0.07 | M01AE01 | ibuprofen | 4 725 | 0.15 | N05BA06 | lorazepam | 31 371 | 1.24 |
| A10AB04 | insulin lispro | 366 | 0.03 | N05BA01 | diazepam | 4 171 | 0.13 | A10BD07 | metformin and sitagliptin | 17 087 | 0.68 |
| N05AX12 | aripiprazole | 339 | 0.03 | N05AH03 | olanzapine | 4 028 | 0.12 | N05BA12 | alprazolam | 14 897 | 0.59 |
| A10AE04 | insulin glargine | 337 | 0.03 | C07AB07 | bisoprolol | 3 835 | 0.12 | N05CD06 | lormetazepam | 14 250 | 0.56 |
| A10AB05 | insulin aspart | 277 | 0.02 | N05AH04 | quetiapine | 3 668 | 0.11 | A10AE04 | insulin glargine | 12 287 | 0.49 |
| L04AX03 | methotrexate | 196 | 0.02 | B01AC06 | acetylsalicylic acid | 3 366 | 0.10 | N02AJ13 | tramadol and paracetamol | 10 822 | 0.43 |
| N05BA01 | diazepam | 177 | 0.01 | N05CD06 | lormetazepam | 3 262 | 0.10 | N05BA01 | diazepam | 10 743 | 0.42 |
| N05BA09 | clobazam | 170 | 0.01 | N05AX08 | risperidone | 3 232 | 0.10 | N05CF02 | zolpidem | 9 560 | 0.38 |
| N05BB01 | hydroxyzine | 135 | 0.01 | A10AB05 | insulin aspart | 3 224 | 0.10 | B01AA07 | acenocoumarol | 9 339 | 0.37 |
| A10AE05 | insulin detemir | 114 | 0.01 | A10AE04 | insulin glargine | 3 129 | 0.10 | A10BD08 | metformin and vildagliptin | 8 904 | 0.35 |
| B01AC06 | acetylsalicylic acid | 98 | 0.01 | N05AX12 | aripiprazole | 3 053 | 0.09 | C03CA01 | furosemide | 8 862 | 0.35 |
| L04AD02 | tacrolimus | 84 | 0.01 | N05AX13 | paliperidone | 3 050 | 0.09 | A10BD15 | metformin and dapagliflozin | 8 445 | 0.33 |
| N05AH03 | olanzapine | 74 | 0.01 | A10BA02 | metformin | 2 559 | 0.08 | C07AB03 | atenolol | 8 434 | 0.33 |
| A10AE06 | insulin degludec | 71 | 0.01 | N05CF02 | zolpidem | 1 918 | 0.06 | A10BD20 | metformin and empagliflozin | 8 274 | 0.33 |
| L04AX01 | azathioprine | 71 | 0.01 | L04AX03 | methotrexate | 1 778 | 0.06 | M01AE01 | ibuprofen | 8 139 | 0.32 |
| H02AB13 | deflazacort | 70 | 0.01 | A10AE06 | insulin degludec | 1 679 | 0.05 | N05AH04 | quetiapine | 7 958 | 0.31 |
| A10AB06 | insulin glulisine | 68 | 0.01 | N05BA05 | potassium clorazepate | 1 586 | 0.05 | A10BK01 | dapagliflozin | 7 636 | 0.30 |
| C07AA05 | propranolol | 59 | 0.00 | A10AB04 | insulin lispro | 1 570 | 0.05 | C07AG02 | carvedilol | 7 502 | 0.30 |
| H02AB07 | prednisone | 56 | 0.00 | N05BA08 | bromazepam | 1 527 | 0.05 | A10BK03 | empagliflozin | 7 476 | 0.30 |
| A10BA02 | metformin | 52 | 0.00 | N02AJ13 | tramadol and paracetamol | 1 520 | 0.05 | L04AX03 | methotrexate | 7 346 | 0.29 |
| N05AX13 | paliperidone | 52 | 0.00 | L04AX01 | azathioprine | 1 441 | 0.04 | N05AH03 | olanzapine | 7 006 | 0.28 |
| N03AF01 | carbamazepine | 49 | 0.00 | M01AE17 | dexketoprofen | 1 381 | 0.04 | N05BA08 | bromazepam | 6 906 | 0.27 |
| L04AA06 | mycophenolic acid | 45 | 0.00 | B01AA07 | acenocoumarol | 1 342 | 0.04 | B01AC04 | clopidogrel | 6 474 | 0.26 |
| H02AB09 | hydrocortisone | 43 | 0.00 | N03AF01 | carbamazepine | 1 189 | 0.04 | C07AB12 | nebivolol | 5 759 | 0.23 |
| C07AB03 | atenolol | 39 | 0.00 | N05AH02 | clozapine | 1 138 | 0.04 | A10BX02 | repaglinide | 5 727 | 0.23 |
| N05CD08 | midazolam | 35 | 0.00 | H02AB07 | prednisone | 1 054 | 0.03 | M01AX25 | chondroitin sulfate | 4 963 | 0.20 |
| B01AA07 | acenocoumarol | 33 | 0.00 | M01AE02 | naproxen | 992 | 0.03 | N03AG01 | valproic acid | 4 954 | 0.20 |

(Table S8 continues on next page)

**Table S8: Prevalence of high-risk medicine use in the general population, stratified by sex and age group.**

(continued from previous page)

| MEN |  |  |  |  |  |  |  |
| --- | --- | --- | --- | --- | --- | --- | --- |
| **65-79 years old** | | | | **80 years and older** | | | |
|  |  | *n* | % |  |  | *n* | % |
| B01AC06 | acetylsalicylic acid | 110 366 | 10.59 | B01AC06 | acetylsalicylic acid | 54 014 | 13.63 |
| C07AB07 | bisoprolol | 64 422 | 6.18 | C03CA01 | furosemide | 31 164 | 7.86 |
| A10BA02 | metformin | 51 788 | 4.97 | C07AB07 | bisoprolol | 29 497 | 7.44 |
| N05BA06 | lorazepam | 28 628 | 2.75 | A10BA02 | metformin | 17 960 | 4.53 |
| C03CA01 | furosemide | 23 593 | 2.26 | N05BA06 | lorazepam | 16 432 | 4.15 |
| A10BD07 | metformin and sitagliptin | 21 721 | 2.08 | B01AA07 | acenocoumarol | 15 364 | 3.88 |
| B01AA07 | acenocoumarol | 20 009 | 1.92 | B01AF02 | apixaban | 10 099 | 2.55 |
| B01AC04 | clopidogrel | 15 471 | 1.48 | B01AC04 | clopidogrel | 9 983 | 2.52 |
| A10AE04 | insulin glargine | 15 121 | 1.45 | C03CA04 | torasemide | 8 462 | 2.14 |
| N05CD06 | lormetazepam | 13 917 | 1.34 | N05CD06 | lormetazepam | 7 723 | 1.95 |
| A10BD08 | metformin and vildagliptin | 13 369 | 1.28 | N02AJ13 | tramadol and paracetamol | 6 893 | 1.74 |
| C07AB03 | atenolol | 12 911 | 1.24 | N05AH04 | quetiapine | 6 115 | 1.54 |
| N02AJ13 | tramadol and paracetamol | 11 971 | 1.15 | A10AE04 | insulin glargine | 6 096 | 1.54 |
| C07AG02 | carvedilol | 11 331 | 1.09 | B01AF01 | rivaroxaban | 6 057 | 1.53 |
| B01AF02 | apixaban | 10 775 | 1.03 | A10BD07 | metformin and sitagliptin | 5 815 | 1.47 |
| A10BX02 | repaglinide | 10 622 | 1.02 | C07AG02 | carvedilol | 5 438 | 1.37 |
| C03CA04 | torasemide | 10 493 | 1.01 | A10BX02 | repaglinide | 5 214 | 1.32 |
| N05CF02 | zolpidem | 9 794 | 0.94 | C03DA01 | spironolactone | 4 985 | 1.26 |
| N05BA12 | alprazolam | 8 706 | 0.84 | A10BH05 | linagliptin | 4 897 | 1.24 |
| A10BK03 | empagliflozin | 8 374 | 0.80 | C07AB03 | atenolol | 4 838 | 1.22 |
| A10BK01 | dapagliflozin | 8 178 | 0.78 | N05CF02 | zolpidem | 4 665 | 1.18 |
| B01AF01 | rivaroxaban | 8 162 | 0.78 | A10BD08 | metformin and vildagliptin | 4 362 | 1.10 |
| N05BA08 | bromazepam | 7 469 | 0.72 | B01AF03 | edoxaban | 3 997 | 1.01 |
| A10BH01 | sitagliptin | 7 323 | 0.70 | N05BA12 | alprazolam | 3 831 | 0.97 |
| A10BD20 | metformin and empagliflozin | 6 863 | 0.66 | N05BA08 | bromazepam | 3 720 | 0.94 |
| A10BD15 | metformin and dapagliflozin | 6 781 | 0.65 | A10BH01 | sitagliptin | 3 524 | 0.89 |
| C03DA01 | spironolactone | 6 452 | 0.62 | C01AA05 | digoxin | 3 202 | 0.81 |
| C07AB12 | nebivolol | 6 265 | 0.60 | B01AE07 | dabigatran etexilate | 2 741 | 0.69 |
| M01AX25 | chondroitin sulfate | 6 067 | 0.58 | H02AB07 | prednisone | 2 630 | 0.66 |
| A10BH05 | linagliptin | 5 784 | 0.56 | C03DA04 | eplerenone | 2 534 | 0.64 |

**Table S9: Most common combinations of chronic diseases at the time of initial diagnosis among people with two or more chronic conditions, stratified by sex and age group.**

| **Age group** | **Women** | | **Men** | |
| --- | --- | --- | --- | --- |
|  | **Chronic disease onset** | **n (%)** | **Chronic disease onset** | **n (%)** |
| **All** | |  |  |  |
|  | Diabetes mellitus + Hypertension | 36 475 (26.20) | Diabetes mellitus + Hypertension | 38 858 (39.99) |
|  | Hypertension + Osteoarthritis | 15 438 (11.09) | Ischaemic heart disease + Hypertension | 6 011 (6.19) |
|  | Hypertension + Osteoporosis | 9 371 (6.73) | Hypertension + Osteoarthritis | 4 729 (4.87) |
|  | Hypertension + Depression | 7 866 (5.65) | Hypertension + COPD | 3 523 (3.63) |
|  | Osteoarthritis + Osteoporosis | 5 467 (3.93) | Hypertension + Malignant neoplasms | 2 962 (3.05) |
| **0-14 years old** | |  |  |  |
|  | Malignant neoplasms + Asthma | 63 (12.02) | Malignant neoplasms + Asthma | 73 (12.41) |
|  | Stroke + Malignant neoplasms | 45 (8.59) | Ischaemic heart disease + Malignant neoplasms | 51 (8.67) |
|  | Ischaemic heart disease + Malignant neoplasms | 40 (7.63) | Stroke + Malignant neoplasms | 47 (7.99) |
|  | Diabetes mellitus + Malignant neoplasms | 31 (5.92) | Dementia + Malignant neoplasms | 35 (5.95) |
|  | Malignant neoplasms + Rheumatoid arthritis | 23 (4.39) | Diabetes mellitus + Malignant neoplasms | 26 (4.42) |
| **15-44 years old** | |  |  |  |
|  | Mental health disorders + Depression | 454 (14.89) | Diabetes mellitus + Hypertension | 533 (17.54) |
|  | Diabetes mellitus + Hypertension | 343 (11.25) | Hypertension + Mental health disorders | 301 (9.90) |
|  | Asthma + Mental health disorders | 244 (8.00) | Hypertension + Asthma | 220 (7.24) |
|  | Hypertension + Mental health disorders | 221 (7.25) | Mental health disorders + Depression | 206 (6.78) |
|  | Asthma + Depression | 188 (6.16) | Asthma + Mental health disorders | 154 (5.07) |
| **45-64 years old** | |  |  |  |
|  | Diabetes mellitus + Hypertension | 5 088 (24.93) | Diabetes mellitus + Hypertension | 10 523 (42.92) |
|  | Hypertension + Depression | 1 393 (6.82) | Hypertension + Mental health disorders | 1 267 (5.17) |
|  | Hypertension + Mental health disorders | 1 390 (6.81) | Ischaemic heart disease + Hypertension | 1 226 (5.00) |
|  | Hypertension + Asthma | 980 (4.80) | Hypertension + Asthma | 925 (3.77) |
|  | Hypertension + Osteoarthritis | 964 (4.72) | Hypertension + Depression | 795 (3.24) |
| **65-79 years old** | |  |  |  |
|  | Diabetes mellitus + Hypertension | 15 329 (29.42) | Diabetes mellitus + Hypertension | 18 445 (44.17) |
|  | Hypertension + Osteoarthritis | 4 942 (9.49) | Ischaemic heart disease + Hypertension | 2 732 (6.54) |
|  | Hypertension + Osteoporosis | 3 745 (7.19) | Hypertension + Osteoarthritis | 1 973 (4.72) |
|  | Hypertension + Depression | 3 430 (6.58) | Hypertension + COPD | 1 545 (3.70) |
|  | Osteoarthritis + Osteoporosis | 2 230 (4.28) | Hypertension + Malignant neoplasms | 1 341 (3.21) |
| **80 years and older** | |  |  |  |
|  | Diabetes mellitus + Hypertension | 15 704 (24.87) | Diabetes mellitus + Hypertension | 9 341 (34.27) |
|  | Hypertension + Osteoarthritis | 9 506 (15.05) | Hypertension + Osteoarthritis | 2 100 (7.70) |
|  | Hypertension + Osteoporosis | 5 217 (8.26) | Ischaemic heart disease + Hypertension | 1 979 (7.26) |
|  | Hypertension + Depression | 2 971 (4.70) | Hypertension + COPD | 1 394 (5.11) |
|  | Osteoarthritis + Osteoporosis | 2 792 (4.42) | Hypertension + Malignant neoplasms | 1 050 (3.85) |

**
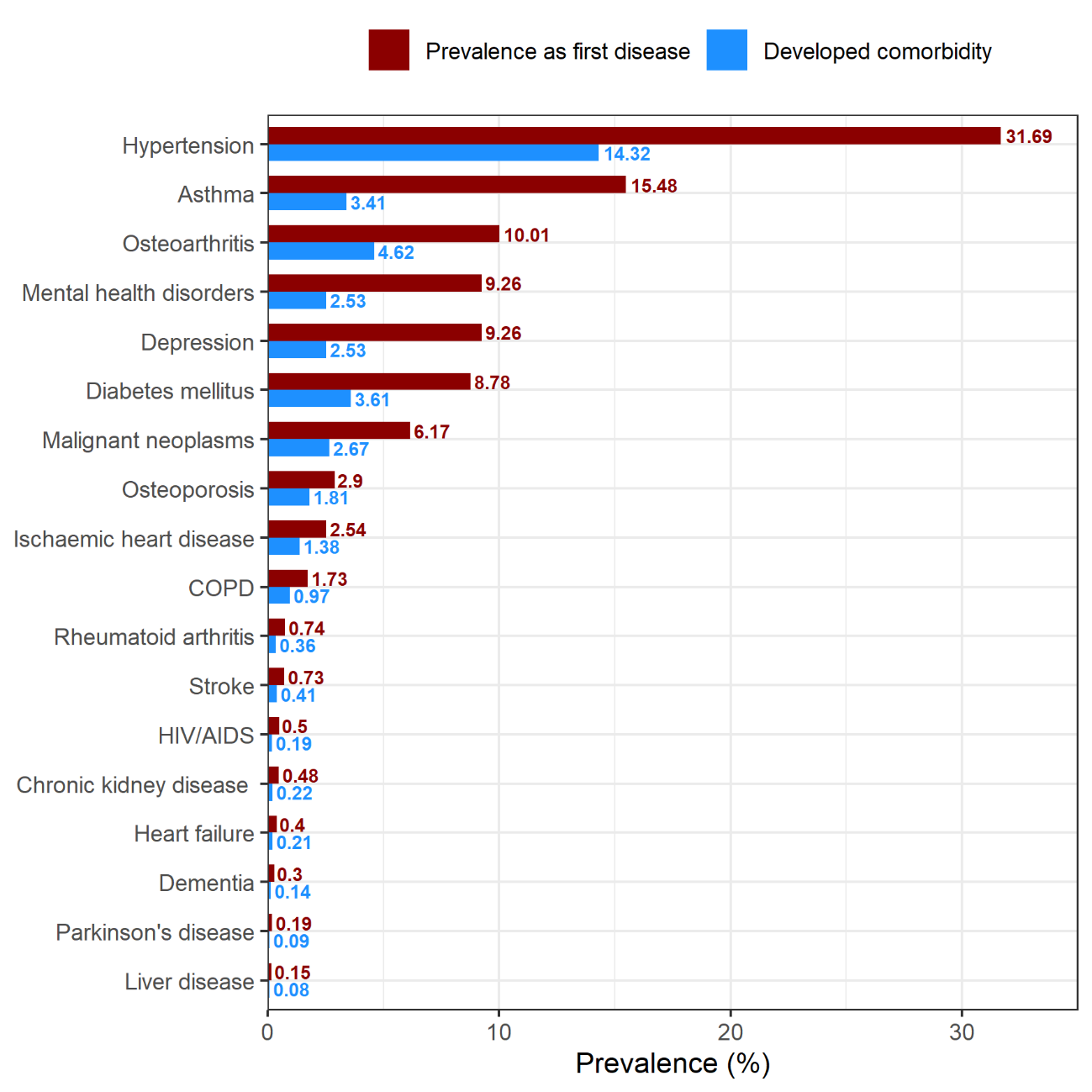
**

**Figure S1. First recorded chronic condition and subsequent comorbidity among incident chronic patients.** For each condition, the bars show the prevalence among individuals who entered chronic care with a single initial diagnosis (n = 6 502 595); the red bar indicates the total prevalence of that condition as the first recorded diagnosis (regardless of whether comorbidity developed), and the blue bar shows the prevalence of those who later developed comorbidity. Mental health disorders refer to chronic mental health conditions other than depression. *COPD* = chronic obstructive pulmonary disease. *HIV/AIDS* = human immunodeficiency virus infection and acquired immune deficiency syndrome.

**Table S10: First recorded chronic condition and subsequent trajectory towards multimorbidity, stratified by sex and age group.**

| First disease | All (*n* = 6 502 595) | | | | |
| --- | --- | --- | --- | --- | --- |
|  | **0-14 years** | **15-44 years** | **45-64 years** | **65-79 years** | **≥80 years** |
| Hypertension | 3 237 (1.08) | 152 212 (11.06) | 780 162 (34.14) | 712 753 (42.61) | 412 084 (47.43) |
| Osteoarthritis | 6 431 (2.14) | 115 279 (8.38) | 223 676 (9.79) | 197 062 (11.78) | 108 577 (12.50) |
| Asthma | 209 851 (69.96) | 512 074 (37.22) | 211 634 (9.26) | 54 465 (3.26) | 18 769 (2.16) |
| Diabetes mellitus | 3921 (1.31) | 49 116 (3.57) | 192 287 (8.41) | 187 673 (11.22) | 85 334 (9.82) |
| Depression | 1962 (0.65) | 155 550 (11.31) | 274 085 (11.99) | 107 055 (6.40) | 32 328 (3.72) |
| Mental health disorders | 37 694 (12.57) | 265 815 (19.32) | 236 949 (10.37) | 49 891 (2.98) | 11 830 (1.36) |
| Malignant neoplasms | 29 140 (9.72) | 69 396 (5.04) | 147 693 (6.46) | 107 267 (6.41) | 47 486 (5.47) |
| Osteoporosis | 59 (0.02) | 1 708 (0.12) | 42 837 (1.87) | 97 497 (5.83) | 46 174 (5.31) |
| Ischaemic heart disease | 3 759 (1.25) | 18 627 (1.35) | 57 966 (2.54) | 55 174 (3.30) | 29 885 (3.44) |
| COPD | 786 (0.26) | 4 921 (0.36) | 40 321 (1.76) | 43 833 (2.62) | 22 478 (2.59) |
| Chronic kidney disease | 203 (0.07) | 2 603 (0.19) | 8 377 (0.37) | 10 753 (0.64) | 9 150 (1.05) |
| Heart failure | 307 (0.10) | 2 216 (0.16) | 5 446 (0.24) | 7 224 (0.43) | 10 593 (1.22) |
| Stroke | 1 095 (0.37) | 4 275 (0.31) | 15 473 (0.68) | 15 199 (0.91) | 11 190 (1.29) |
| Dementia | 123 (0.04) | 268 (0.02) | 1 324 (0.06) | 4 979 (0.30) | 12 820 (1.48) |
| Rheumatoid arthritis | 869 (0.29) | 8 440 (0.61) | 20 924 (0.92) | 12 549 (0.75) | 5 137 (0.59) |
| Parkinson's disease | 193 (0.06) | 642 (0.05) | 2 584 (0.11) | 4 866 (0.29) | 4 214 (0.48) |
| HIV/AIDS | 268 (0.09) | 11 980 (0.87) | 18 431 (0.81) | 1 504 (0.09) | 118 (0.01) |
| Liver disease | 48 (0.02) | 798 (0.06) | 5 008 (0.22) | 2 923 (0.17) | 718 (0.08) |
|  |  |  |  |  |  |
| Evolved to multimorbidity | All (*n* = 2 686 457) | | | | |
|  | **0-14 years** | **15-44 years** | **45-64 years** | **65-79 years** | **≥80 years** |
| Hypertension | 206 (6.36) | 19 494 (12.81) | 224 801 (28.81) | 388 522 (54.51) | 298 427 (72.42) |
| Osteoarthritis | 326 (5.07) | 18 753 (16.27) | 75 601 (33.80) | 120 073 (60.93) | 85 418 (78.67) |
| Asthma | 6 896 (3.29) | 75 445 (14.73) | 84 189 (39.78) | 39 164 (71.91) | 16 121 (85.89) |
| Diabetes mellitus | 214 (5.46) | 10 015 (20.39) | 80 480 (41.85) | 121 950 (64.98) | 67 553 (79.16) |
| Depression | 105 (5.35) | 25 528 (16.41) | 108 116 (39.45) | 75 056 (70.11) | 26 230 (81.14) |
| Mental health disorders | 3 927 (10.42) | 37 940 (14.27) | 82 605 (34.86) | 31 342 (62.82) | 8 665 (73.25) |
| Malignant neoplasms | 2 425 (8.32) | 12 929 (18.63) | 54 552 (36.94) | 66 519 (62.01) | 36 922 (77.75) |
| Osteoporosis | ≤10 (≤16.95) | 347 (20.32) | 15 273 (35.65) | 63 237 (64.86) | 38 775 (83.98) |
| Ischaemic heart disease | 509 (13.54) | 3 773 (20.26) | 24 226 (41.79) | 36 682 (66.48) | 24 234 (81.09) |
| COPD | 125 (15.90) | 1 177 (23.92) | 16 461 (40.82) | 27 621 (63.01) | 17 511 (77.90) |
| Chronic kidney disease | 28 (13.79) | 540 (20.75) | 3 092 (36.91) | 5 315 (49.43) | 5 363 (58.61) |
| Heart failure | 44 (14.33) | 369 (16.65) | 2 270 (41.68) | 4 321 (59.81) | 6 421 (60.62) |
| Stroke | 113 (10.32) | 950 (22.22) | 7 109 (45.94) | 10 146 (66.75) | 8 226 (73.51) |
| Dementia | ≤10 (≤8.13) | 55 (20.52) | 412 (31.12) | 2 077 (41.72) | 6 340 (49.45) |
| Rheumatoid arthritis | 46 (5.29) | 1 545 (18.31) | 8 669 (41.43) | 8 846 (70.49) | 4 231 (82.36) |
| Parkinson's disease | 15 (7.77) | 114 (17.76) | 818 (31.66) | 2 421 (49.75) | 2 734 (64.88) |
| HIV/AIDS | 27 (10.07) | 1 812 (15.13) | 9 309 (50.51) | 1 082 (71.94) | 99 (83.90) |
| Liver disease | ≤10 (≤20.83) | 133 (16.67) | 2 256 (45.05) | 2 033 (69.55) | 594 (82.73) |

(Table S10 continues on next page)

**Table S10: First recorded chronic condition and subsequent trajectory towards multimorbidity, stratified by sex and age group.**

(continued from previous page)

| First disease | Women (*n* = 3 540 859) | | | | |
| --- | --- | --- | --- | --- | --- |
|  | **0-14 years** | **15-44 years** | **45-64 years** | **65-79 years** | **≥80 years** |
| Hypertension | 1 538 (1.18) | 59 689 (8.27) | 327 675 (26.93) | 362 467 (39.32) | 266 028 (48.27) |
| Osteoarthritis | 2 975 (2.29) | 61 304 (8.50) | 149 904 (12.32) | 134 996 (14.64) | 77 038 (13.98) |
| Asthma | 85 067 (65.51) | 246 786 (34.21) | 127 557 (10.48) | 35 857 (3.89) | 13 442 (2.44) |
| Diabetes mellitus | 1 890 (1.46) | 21 996 (3.05) | 65 937 (5.42) | 72 236 (7.84) | 45 831 (8.32) |
| Depression | 1 061 (0.82) | 105 107 (14.57) | 195 352 (16.06) | 80 656 (8.75) | 25 386 (4.61) |
| Mental health disorders | 19 540 (15.05) | 160 927 (22.31) | 149 251 (12.27) | 34 042 (3.69) | 8 648 (1.57) |
| Malignant neoplasms | 14 061 (10.83) | 42 280 (5.86) | 97 629 (8.02) | 55 597 (6.03) | 23 226 (4.21) |
| Osteoporosis | 24 (0.02) | 1 159 (0.16) | 40 359 (3.32) | 94 461 (10.25) | 44 204 (8.02) |
| Ischaemic heart disease | 1 745 (1.34) | 7 293 (1.01) | 13 744 (1.13) | 12 693 (1.38) | 9 977 (1.81) |
| COPD | 359 (0.28) | 1 981 (0.27) | 14 808 (1.22) | 10 497 (1.14) | 4 949 (0.90) |
| Chronic kidney disease | 62 (0.05) | 929 (0.13) | 3 262 (0.27) | 4 394 (0.48) | 4 891 (0.89) |
| Heart failure | 140 (0.11) | 678 (0.09) | 1 778 (0.15) | 3 191 (0.35) | 6 465 (1.17) |
| Stroke | 500 (0.39) | 2 041 (0.28) | 5 983 (0.49) | 5 472 (0.59) | 5 449 (0.99) |
| Dementia | 64 (0.05) | 128 (0.02) | 616 (0.05) | 2 916 (0.32) | 9 143 (1.66) |
| Rheumatoid arthritis | 566 (0.44) | 6 139 (0.85) | 15 270 (1.26) | 8 905 (0.97) | 3 799 (0.69) |
| Parkinson's disease | 104 (0.08) | 320 (0.04) | 982 (0.08) | 1 972 (0.21) | 2 224 (0.4) |
| HIV/AIDS | 125 (0.10) | 2 173 (0.30) | 4 481 (0.37) | 277 (0.03) | 29 (0.01) |
| Liver disease | 23 (0.02) | 400 (0.06) | 2 018 (0.17) | 1 314 (0.14) | 407 (0.07) |
|  |  |  |  |  |  |
| Evolved to multimorbidity | Women (*n* = 1 590 017) | | | | |
|  | **0-14 years** | **15-44 years** | **45-64 years** | **65-79 years** | **≥80 years** |
| Hypertension | 83 (5.40) | 8 808 (14.76) | 105 522 (32.20) | 211 585 (58.37) | 199 413 (74.96) |
| Osteoarthritis | 158 (5.31) | 11 080 (18.07) | 51 427 (34.31) | 83 873 (62.13) | 62 141 (80.66) |
| Asthma | 2 785 (3.27) | 40 648 (16.47) | 54 656 (42.85) | 26 692 (74.44) | 11 757 (87.46) |
| Diabetes mellitus | 96 (5.08) | 4 960 (22.55) | 29 660 (44.98) | 49 320 (68.28) | 37 005 (80.74) |
| Depression | 43 (4.05) | 17 756 (16.89) | 78 811 (40.34) | 57 627 (71.45) | 21 109 (83.15) |
| Mental health disorders | 1 867 (9.55) | 23 467 (14.58) | 52 759 (35.35) | 21 860 (64.21) | 6 484 (74.98) |
| Malignant neoplasms | 1 034 (7.35) | 8 036 (19.01) | 37 381 (38.29) | 37 836 (68.05) | 19 384 (83.46) |
| Osteoporosis | ≤10 (≤41.67) | 238 (20.53) | 14 234 (35.27) | 61 266 (64.86) | 37 238 (84.24) |
| Ischaemic heart disease | 203 (11.63) | 1 591 (21.82) | 6 452 (46.94) | 9 037 (71.20) | 8 472 (84.92) |
| COPD | 65 (18.11) | 535 (27.01) | 6 392 (43.17) | 6 628 (63.14) | 4 055 (81.94) |
| Chronic kidney disease | ≤10 (≤16.13) | 194 (20.88) | 1 221 (37.43) | 2 194 (49.93) | 2 857 (58.41) |
| Heart failure | 12 (8.57) | 123 (18.14) | 749 (42.13) | 1 952 (61.17) | 3 980 (61.56) |
| Stroke | 51 (10.20) | 470 (23.03) | 2 806 (46.90) | 3 783 (69.13) | 4 054 (74.40) |
| Dementia | ≤10 (≤15.63) | 27 (21.09) | 191 (31.01) | 1 267 (43.45) | 4 596 (50.27) |
| Rheumatoid arthritis | 28 (4.95) | 1 177 (19.17) | 6 396 (41.89) | 6 423 (72.13) | 3 200 (84.23) |
| Parkinson's disease | ≤10 (≤9.62) | 66 (20.63) | 343 (34.93) | 1 105 (56.03) | 1 554 (69.87) |
| HIV/AIDS | ≤10 (≤8.00) | 485 (22.32) | 2 634 (58.78) | 224 (80.87) | 27 (93.10) |
| Liver disease | ≤10 (≤43.48) | 63 (15.75) | 895 (44.35) | 949 (72.22) | 357 (87.71) |

(Table S10 continues on next page)

**Table S10: First recorded chronic condition and subsequent trajectory towards multimorbidity, stratified by sex and age group.**

(continued from previous page)

| First disease | Men (*n* = 2 961 736) | | | | |
| --- | --- | --- | --- | --- | --- |
|  | **0-14 years** | **15-44 years** | **45-64 years** | **65-79 years** | **≥80 years** |
| Hypertension | 1 699 (1.00) | 92 523 (14.13) | 452 487 (42.35) | 350 286 (46.66) | 146 056 (45.97) |
| Osteoarthritis | 3 456 (2.03) | 53 975 (8.25) | 73 772 (6.90) | 62 066 (8.27) | 31 539 (9.93) |
| Asthma | 124 784 (73.36) | 265 288 (40.53) | 84 077 (7.87) | 18 608 (2.48) | 5 327 (1.68) |
| Diabetes mellitus | 2 031 (1.19) | 27 120 (4.14) | 126 350 (11.82) | 115 437 (15.38) | 39 503 (12.43) |
| Depression | 901 (0.53) | 50 443 (7.71) | 78 733 (7.37) | 26 399 (3.52) | 6 942 (2.18) |
| Mental health disorders | 18 154 (10.67) | 104 888 (16.02) | 87 698 (8.21) | 15 849 (2.11) | 3 182 (1.00) |
| Malignant neoplasms | 15 079 (8.86) | 27 116 (4.14) | 50 064 (4.69) | 51 670 (6.88) | 24 260 (7.63) |
| Osteoporosis | 35 (0.02) | 549 (0.08) | 2 478 (0.23) | 3 036 (0.40) | 1 970 (0.62) |
| Ischaemic heart disease | 2 014 (1.18) | 11 334 (1.73) | 44 222 (4.14) | 42 481 (5.66) | 19 908 (6.27) |
| COPD | 427 (0.25) | 2 940 (0.45) | 25 513 (2.39) | 33 336 (4.44) | 17 529 (5.52) |
| Chronic kidney disease | 141 (0.08) | 1 674 (0.26) | 5 115 (0.48) | 6 359 (0.85) | 4 259 (1.34) |
| Heart failure | 167 (0.10) | 1 538 (0.23) | 3 668 (0.34) | 4 033 (0.54) | 4 128 (1.30) |
| Stroke | 595 (0.35) | 2 234 (0.34) | 9 490 (0.89) | 9 727 (1.30) | 5 741 (1.81) |
| Dementia | 59 (0.03) | 140 (0.02) | 708 (0.07) | 2 063 (0.27) | 3 677 (1.16) |
| Rheumatoid arthritis | 303 (0.18) | 2 301 (0.35) | 5 654 (0.53) | 3 644 (0.49) | 1 338 (0.42) |
| Parkinson's disease | 89 (0.05) | 322 (0.05) | 1 602 (0.15) | 2 894 (0.39) | 1 990 (0.63) |
| HIV/AIDS | 143 (0.08) | 9 807 (1.50) | 13 950 (1.31) | 1 227 (0.16) | 89 (0.03) |
| Liver disease | 25 (0.01) | 398 (0.06) | 2 990 (0.28) | 1 609 (0.21) | 311 (0.10) |
|  |  |  |  |  |  |
| Evolved to multimorbidity | Men (*n* = 1 096 440) | | | | |
|  | **0-14 years** | **15-44 years** | **45-64 years** | **65-79 years** | **≥80 years** |
| Hypertension | 123 (7.24) | 10 686 (11.55) | 119 279 (26.36) | 176 937 (50.51) | 99 014 (67.79) |
| Osteoarthritis | 168 (4.86) | 7 673 (14.22) | 24 174 (32.77) | 36 200 (58.33) | 23 277 (73.80) |
| Asthma | 4 111 (3.29) | 34 797 (13.12) | 29 533 (35.13) | 12 472 (67.02) | 4 364 (81.92) |
| Diabetes mellitus | 118 (5.81) | 5 055 (18.64) | 50 820 (40.22) | 72 630 (62.92) | 30 548 (77.33) |
| Depression | 62 (6.88) | 7 772 (15.41) | 29 305 (37.22) | 17 429 (66.02) | 5 121 (73.77) |
| Mental health disorders | 2 060 (11.35) | 14 473 (13.80) | 29 846 (34.03) | 9 482 (59.83) | 2 181 (68.54) |
| Malignant neoplasms | 1 391 (9.22) | 4 893 (18.04) | 17 171 (34.30) | 28 683 (55.51) | 17 538 (72.29) |
| Osteoporosis | ≤10 (≤28.57) | 109 (19.85) | 1 039 (41.93) | 1 971 (64.92) | 1 537 (78.02) |
| Ischaemic heart disease | 306 (15.19) | 2 182 (19.25) | 17 774 (40.19) | 27 645 (65.08) | 15 762 (79.17) |
| COPD | 60 (14.05) | 642 (21.84) | 10 069 (39.47) | 20 993 (62.97) | 13 456 (76.76) |
| Chronic kidney disease | 21 (14.89) | 346 (20.67) | 1 871 (36.58) | 3 121 (49.08) | 2 506 (58.84) |
| Heart failure | 32 (19.16) | 246 (15.99) | 1 521 (41.47) | 2 369 (58.74) | 2 441 (59.13) |
| Stroke | 62 (10.42) | 480 (21.49) | 4 303 (45.34) | 6 363 (65.42) | 4 172 (72.67) |
| Dementia | ≤10 (≤16.95) | 28 (20.00) | 221 (31.21) | 810 (39.26) | 1 744 (47.43) |
| Rheumatoid arthritis | 18 (5.94) | 368 (15.99) | 2 273 (40.20) | 2 423 (66.49) | 1 031 (77.06) |
| Parkinson's disease | ≤10 (≤11.24) | 48 (14.91) | 475 (29.65) | 1 316 (45.47) | 1 180 (59.30) |
| HIV/AIDS | 17 (11.89) | 1 327 (13.53) | 6 675 (47.85) | 858 (69.93) | 72 (80.90) |
| Liver disease | ≤10 (≤40.00) | 70 (17.59) | 1 361 (45.52) | 1 084 (67.37) | 237 (76.21) |

**Table S11: Most frequent trajectories of multimorbidity onset in women by age group; progression to ≥3 conditions, polypharmacy, high-risk medicine use, and exposure to medicines with anticholinergic activity.**

| **Age group** | **Trajectory onset (First disease → Second disease)** | **n (% prevalence)^*^** | **Additional comorbidity^†^** | **Polypharmacy^‡^** | **High-risk medicines^§^** | **Medicines with anticholinergic activity^▲^** |
| --- | --- | --- | --- | --- | --- | --- |
| **0-14 years old** | |  |  |  |  |  |
|  | Mental health disorders → Asthma | 1 499 (21.46) | 50 (3.34) | 0 (0.00) | 106 (7.07) | 29 (1.93) |
|  | Asthma → Mental health disorders | 1 086 (15.55) | 30 (2.76) | 0 (0.00) | 54 (4.97) | 28 (2.58) |
|  | Malignant neoplasms → Asthma | 778 (11.14) | 18 (2.37) | 0 (0.00) | 57 (7.33) | 14 (1.80) |
|  | Asthma → Malignant neoplasms | 517 (7.40) | 11 (2.13) | ≤10 (≤3.68) | 43 (8.32) | 17 (3.29) |
|  | Asthma → Osteoarthritis | 508 (7.27) | 12 (2.36) | 0 (0.00) | 21 (4.13) | 15 (2.95) |
| **15-44 years old** | |  |  |  |  |  |
|  | Asthma → Mental health disorders | 13 400 (10.91) | 1 580 (11.79) | 140 (1.04) | 875 (6.53) | 859 (6.41) |
|  | Asthma → Depression | 9 233 (7.52) | 1 265 (13.70) | 155 (1.68) | 804 (8.71) | 822 (8.90) |
|  | Mental health disorders → Depression | 8 004 (6.52) | 728 (9.10) | 164 (2.05) | 1 209 (15.10) | 1 219 (15.23) |
|  | Asthma → Osteoarthritis | 6 968 (5.68) | 1 007 (14.45) | 42 (0.60) | 304 (4.36) | 265 (3.80) |
|  | Depression → Mental health disorders | 6 237 (5.08) | 706 (11.32) | 118 (1.89) | 792 (12.70) | 764 (12.25) |
|  | Mental health disorders → Asthma | 5 813 (4.73) | 720 (12.39) | 96 (1.65) | 495 (8.52) | 491 (8.45) |
|  | Asthma → Hypertension | 4 773 (3.89) | 577 (12.09) | 136 (2.85) | 414 (8.67) | 309 (6.47) |
| **45-64 years old** | |  |  |  |  |  |
|  | Depression → Hypertension | 24 427 (5.16) | 6 773 (27.73) | 2 385 (9.76) | 6 517 (26.68) | 5 168 (21.16) |
|  | Hypertension → Osteoarthritis | 22 504 (4.76) | 5 616 (24.96) | 2 429 (10.79) | 6 194 (27.52) | 3 352 (14.90) |
|  | Hypertension → Diabetes mellitus | 19 215 (4.06) | 4 876 (25.38) | 4 299 (22.37) | 9 678 (50.37) | 2 991 (15.57) |
|  | Osteoarthritis → Hypertension | 18 610 (3.93) | 4 053 (21.78) | 1 478 (7.94) | 4 158 (22.34) | 2 546 (13.68) |
|  | Hypertension → Depression | 16 783 (3.55) | 5 255 (31.31) | 2 322 (13.84) | 5 278 (31.45) | 3 880 (23.12) |
|  | Mental health disorders → Hypertension | 16 296 (3.45) | 3 826 (23.48) | 1 225 (7.52) | 3 712 (22.78) | 2 772 (17.01) |
|  | Depression → Osteoarthritis | 15 760 (3.33) | 4 694 (29.78) | 1 055 (6.69) | 3 774 (23.95) | 3 152 (20.00) |
|  | Asthma → Hypertension | 15 704 (3.32) | 4 644 (29.75) | 1 336 (8.51) | 2 961 (18.86) | 2 051 (13.06) |
| **65-79 years old** | |  |  |  |  |  |
|  | Hypertension → Osteoarthritis | 59 612 (9.38) | 24 805 (41.61) | 12 056 (20.22) | 22 699 (38.08) | 11 552 (19.38) |
|  | Hypertension → Diabetes mellitus | 37 566 (5.91) | 17 039 (45.36) | 12 632 (33.63) | 21 453 (57.11) | 7 330 (19.51) |
|  | Osteoarthritis → Hypertension | 37 144 (5.84) | 13 999 (37.69) | 6 227 (16.76) | 12 604 (33.93) | 6 669 (17.95) |
|  | Diabetes mellitus → Hypertension | 25 147 (3.96) | 11 511 (45.77) | 10 287 (40.91) | 16 383 (65.15) | 5 420 (21.55) |
|  | Hypertension → Depression | 25 037 (3.94) | 13 562 (54.17) | 5 821 (23.25) | 10 105 (40.36) | 6 872 (27.45) |
|  | Hypertension → Osteoporosis | 24 329 (3.83) | 12 805 (52.63) | 5 220 (21.46) | 8 870 (36.46) | 4 584 (18.84) |
|  | Osteoporosis → Hypertension | 21 458 (3.38) | 8 936 (41.64) | 3 126 (14.57) | 6 318 (29.44) | 3 344 (15.58) |
|  | Depression → Hypertension | 19 996 (3.15) | 9 490 (47.46) | 3 853 (19.27) | 7 337 (36.69) | 5 345 (26.73) |
| **80 years and older** | |  |  |  |  |  |
|  | Hypertension → Osteoarthritis | 62 146 (12.66) | 36 742 (59.12) | 16 724 (26.91) | 28 533 (45.91) | 14 123 (22.73) |
|  | Osteoarthritis → Hypertension | 30 132 (6.14) | 17 022 (56.49) | 7 828 (25.98) | 13 397 (44.46) | 6 864 (22.78) |
|  | Hypertension → Diabetes mellitus | 28 250 (5.76) | 17 712 (62.70) | 10 395 (36.8) | 16 170 (57.24) | 5 979 (21.16) |
|  | Hypertension → Osteoporosis | 23 351 (4.76) | 16 099 (68.94) | 6 642 (28.44) | 10 543 (45.15) | 5 175 (22.16) |
|  | Diabetes mellitus → Hypertension | 18 917 (3.85) | 12 024 (63.56) | 8 219 (43.45) | 11 942 (63.13) | 4 349 (22.99) |
|  | Hypertension → Depression | 18 223 (3.71) | 11 700 (64.20) | 5 398 (29.62) | 8 607 (47.23) | 5 400 (29.63) |
|  | Osteoporosis → Hypertension | 14 990 (3.05) | 9 181 (61.25) | 3 656 (24.39) | 6 263 (41.78) | 3 166 (21.12) |

Trajectories of multimorbidity onset with prevalence ≥3% among people with multimorbidity (≥2 chronic conditions) in the corresponding age group. Data are n (%), unless stated. Values ≤10, except zero, shown as “≤10”. ^*^n (% prevalence): Prevalence within multimorbidity population in the age group. ^†^Additional comorbidity: ≥1 further chronic condition (third or more). ^‡^Polypharmacy: ≥5 concurrent medicines for ≥6 months (≤30-day gaps). ^§^High-risk medicine: ≥1 medicine classified as high-risk for chronic patients. ^▲^Anticholinergic activity: ≥1 medicine with known anticholinergic properties.

**Table S12: Most frequent trajectories of multimorbidity onset in men by age group; progression to ≥3 conditions, polypharmacy, high-risk medicine use, and exposure to medicines with anticholinergic activity.**

| **Age group** | **Trajectory onset (First disease → Second disease)** | **n (% prevalence) ^*^** | **Additional comorbidity^†^** | **Polypharmacy^‡^** | **High-risk medicines^§^** | **Medicines with anticholinergic activity^▲^** |
| --- | --- | --- | --- | --- | --- | --- |
| **0-14 years old** | |  |  |  |  |  |
|  | Mental health disorders → Asthma | 1 733 (18.93) | 45 (2.60) | ≤10 (≤0.58) | 84 (4.85) | 36 (2.08) |
|  | Asthma → Mental health disorders | 1 505 (16.44) | 33 (2.19) | ≤10 (≤0.66) | 67 (4.45) | 32 (2.13) |
|  | Malignant neoplasms → Asthma | 1 149 (12.55) | 28 (2.44) | ≤10 (≤0.87) | 83 (7.22) | 31 (2.70) |
|  | Asthma → Malignant neoplasms | 866 (9.46) | 21 (242) | ≤10 (≤1.15) | 56 (6.47) | 23 (2.66) |
|  | Asthma → Osteoarthritis | 769 (8.40) | 19 (2.47) | 0 (0.00) | 29 (3.77) | ≤10 (≤1.30) |
|  | Asthma → Hypertension | 311 (3.40) | 12 (3.86) | 0 (0.00) | 12 (3.86) | 14 (4.50) |
| **15-44 years old** | |  |  |  |  |  |
|  | Asthma → Mental health disorders | 9 229 (9.79) | 913 (9.89) | 76 (0.82) | 527 (5.71) | 529 (5.73) |
|  | Asthma → Osteoarthritis | 7 134 (7.57) | 728 (10.20) | 11 (0.15) | 134 (1.88) | 122 (1.71) |
|  | Asthma → Hypertension | 6 608 (7.01) | 614 (9.29) | 87 (1.32) | 351 (5.31) | 240 (3.63) |
|  | Asthma → Depression | 4 690 (4.98) | 572 (12.20) | 42 (0.90) | 304 (6.48) | 309 (6.59) |
|  | Mental health disorders → Depression | 3 851 (4.09) | 311 (8.08) | 160 (4.15) | 924 (23.99) | 803 (20.85) |
|  | Mental health disorders → Hypertension | 3 502 (3.72) | 302 (8.62) | 109 (3.11) | 527 (15.05) | 444 (12.68) |
|  | Mental health disorders → Asthma | 3 458 (3.67) | 383 (11.08) | 59 (1.71) | 299 (8.65) | 280 (8.10) |
| **45-64 years old** | |  |  |  |  |  |
|  | Hypertension → Diabetes mellitus | 39 078 (10.50) | 7 587 (19.42) | 6 592 (16.87) | 18 457 (47.23) | 2 995 (7.66) |
|  | Diabetes mellitus → Hypertension | 30 590 (8.22) | 6 377 (20.85) | 6 456 (21.1) | 16 509 (53.97) | 2 384 (7.79) |
|  | Hypertension → Osteoarthritis | 15 102 (4.06) | 3 247 (21.50) | 1 319 (8.73) | 3 750 (24.83) | 1 435 (9.50) |
|  | Asthma → Hypertension | 13 502 (3.63) | 2 811 (20.82) | 726 (5.38) | 1 896 (14.04) | 934 (6.92) |
|  | Depression → Hypertension | 12 471 (3.35) | 2 878 (23.08) | 1 069 (8.57) | 2 976 (23.86) | 2 038 (16.34) |
|  | Mental health disorders → Hypertension | 12 401 (3.33) | 2 581 (20.81) | 922 (7.43) | 2 872 (23.16) | 2 000 (16.13) |
|  | Osteoarthritis → Hypertension | 11 893 (3.20) | 2 431 (20.44) | 796 (6.69) | 2 465 (20.73) | 951 (8.00) |
|  | Hypertension → Ischaemic heart disease | 11 517 (3.09) | 3 368 (29.24) | 2 699 (23.43) | 5 509 (47.83) | 1 202 (10.44) |
| **65-79 years old** | |  |  |  |  |  |
|  | Hypertension → Diabetes mellitus | 51 264 (11.04) | 19 091 (37.24) | 14 830 (28.93) | 28 036 (54.69) | 5 055 (9.86) |
|  | Diabetes mellitus → Hypertension | 40 662 (8.75) | 15 279 (37.58) | 13 518 (33.24) | 24 591 (60.48) | 4 132 (10.16) |
|  | Hypertension → Osteoarthritis | 30 363 (6.54) | 11 276 (37.14) | 5 481 (18.05) | 10 934 (36.01) | 3 493 (11.50) |
|  | Hypertension → Malignant neoplasms | 22 986 (4.95) | 7 542 (32.81) | 3 457 (15.04) | 7 080 (30.80) | 2 246 (9.77) |
|  | Osteoarthritis → Hypertension | 17 954 (3.86) | 6 364 (35.45) | 2 864 (15.95) | 5 958 (33.18) | 1 939 (10.80) |
|  | Hypertension → Ischaemic heart disease | 17 118 (3.68) | 7 718 (45.09) | 5 646 (32.98) | 9 243 (54.00) | 2 219 (12.96) |
| **80 years and older** | |  |  |  |  |  |
|  | Hypertension → Osteoarthritis | 20 718 (8.17) | 10 995 (53.07) | 5 735 (27.68) | 9405 (45.40) | 3 322 (16.03) |
|  | Hypertension → Diabetes mellitus | 19 005 (7.50) | 10 636 (55.96) | 6 583 (34.64) | 10 514 (55.32) | 2 469 (12.99) |
|  | Hypertension → Malignant neoplasms | 13 375 (5.28) | 6 632 (49.59) | 3 105 (23.21) | 5 290 (39.55) | 1 779 (13.30) |
|  | Hypertension → Ischaemic heart disease | 9 139 (3.61) | 5 547 (60.70) | 3 363 (36.80) | 5 104 (55.85) | 1 482 (16.22) |

Trajectories of multimorbidity onset with prevalence ≥3% among people with multimorbidity (≥2 chronic conditions) in the corresponding age group. Data are n (%), unless stated. Values ≤10, except zero, shown as “≤10”. ^*^n (% prevalence): Prevalence within multimorbidity population in the age group. All other percentages indicate the proportion of people within that trajectory presenting the corresponding feature. ^†^Additional comorbidity: ≥1 further chronic condition (third or more). ^‡^Polypharmacy: ≥5 concurrent medicines for ≥6 months (≤30-day gaps). ^§^High-risk medicine: ≥1 medicine classified as high-risk for chronic patients. ^▲^Anticholinergic activity: ≥1 medicine with anticholinergic activity.

**Table S13: Most common trajectories of multimorbidity onset originating from each of the 18 chronic conditions in women.**

| First disease | Second disease | n (% prevalence)^*^ | Additional comorbidity^†^ | Polypharmacy^‡^ | High-risk medicines^§^ | Medicines with anticholinergic activity^▲^ |
| --- | --- | --- | --- | --- | --- | --- |
| Hypertension | Osteoarthritis | 145 077 (8.38) | 67 295 (46.39) | 31 237 (21.53) | 57 535 (39.66) | 29 103 (20.06) |
|  | Diabetes mellitus | 86 131 (4.98) | 39 730 (46.13) | 27 444 (31.86) | 47 713 (55.40) | 16 407 (19.05) |
|  | Depression | 61 896 (3.57) | 30 763 (49.7) | 13 630 (22.02) | 24 325 (39.30) | 16 424 (26.53) |
| Asthma | Hypertension | 34 392 (1.99) | 13 423 (39.03) | 4 775 (13.88) | 8426 (24.50) | 5 398 (15.70) |
|  | Osteoarthritis | 23 878 (1.38) | 8 652 (36.23) | 2 039 (8.54) | 4 318 (18.08) | 2 949 (12.35) |
|  | Mental health disorders | 23 410 (1.35) | 5 108 (21.82) | 740 (3.16) | 2 449 (10.46) | 2 157 (9.21) |
| Osteoarthritis | Hypertension | 87 251 (5.04) | 35 249 (40.40) | 15 553 (17.83) | 30 293 (34.72) | 16 166 (18.53) |
|  | Depression | 25 989 (1.50) | 11 698 (45.01) | 3 790 (14.58) | 8 444 (32.49) | 6 120 (23.55) |
|  | Osteoporosis | 23 028 (1.33) | 12 743 (55.34) | 3 624 (15.74) | 7 724 (33.54) | 4 282 (18.59) |
| Mental health disorders | Hypertension | 30 233 (1.75) | 8 611 (28.48) | 3 490 (11.54) | 8 233 (27.23) | 5 922 (19.59) |
|  | Depression | 21 937 (1.27) | 5 110 (23.29) | 1 380 (6.29) | 5 157 (23.51) | 4 670 (21.29) |
|  | Osteoarthritis | 17 254 (1.00) | 5 801 (33.62) | 1 330 (7.71) | 3 989 (23.12) | 3 034 (17.58) |
| Depression | Hypertension | 54 808 (3.16) | 21 481 (39.19) | 8 571 (15.64) | 17 916 (32.69) | 13 350 (24.36) |
|  | Osteoarthritis | 36 095 (2.09) | 15 507 (42.96) | 4 502 (12.47) | 11 025 (30.54) | 8 642 (23.94) |
|  | Mental health disorders | 19 692 (1.14) | 5 411 (27.48) | 1 051 (5.34) | 3 960 (20.11) | 3 508 (17.81) |
| Diabetes mellitus | Hypertension | 59 558 (3.44) | 27 569 (46.29) | 22 216 (37.30) | 36 566 (61.40) | 12 101 (20.32) |
|  | Osteoarthritis | 16 946 (0.98) | 9 387 (55.39) | 5 804 (34.25) | 10 378 (61.24) | 3 660 (21.60) |
|  | Depression | 9 286 (0.54) | 4 886 (52.62) | 2 942 (31.68) | 5 362 (57.74) | 2 502 (26.94) |
| Malignant neoplasms | Hypertension | 32 760 (1.89) | 15 111 (46.13) | 5 640 (17.22) | 11 128 (33.97) | 5 759 (17.58) |
|  | Osteoarthritis | 15 390 (0.89) | 7 370 (47.89) | 1 858 (12.07) | 4 535 (29.47) | 2 517 (16.35) |
|  | Depression | 15 252 (0.88) | 6 778 (44.44) | 1 732 (11.36) | 4 386 (28.76) | 3 402 (22.31) |
| Osteoporosis | Hypertension | 40 322 (2.33) | 19 142 (47.47) | 7 172 (17.79) | 13 502 (33.49) | 7 061 (17.51) |
|  | Osteoarthritis | 28 200 (1.63) | 14 830 (52.59) | 3 864 (13.70) | 8 621 (30.57) | 4 766 (16.90) |
|  | Depression | 12 953 (0.75) | 7 235 (55.86) | 2 086 (16.10) | 4 368 (33.72) | 3 184 (24.58) |
| Ischaemic heart disease | Hypertension | 8 375 (0.48) | 4 691 (56.01) | 2 414 (28.82) | 3 958 (47.26) | 1 781 (21.27) |
|  | Diabetes mellitus | 3 956 (0.23) | 2 157 (54.52) | 946 (23.91) | 1 669 (42.19) | 809 (20.45) |
|  | Depression | 2 636 (0.15) | 1 358 (51.52) | 569 (21.59) | 998 (37.86) | 673 (25.53) |
| COPD | Hypertension | 5 325 (0.31) | 2 545 (47.79) | 1 456 (27.34) | 147 (39.91) | 1 303 (24.47) |
|  | Osteoarthritis | 2 421 (0.14) | 1 280 (52.87) | 538 (22.22) | 155 (38.29) | 599 (24.74) |
|  | Asthma | 1 883 (0.11) | 951 (50.50) | 452 (24.00) | 96 (34.73) | 478 (25.39) |

(Table S13 continues on next page)

**Table S13: Most common trajectories of multimorbidity onset originating from each of the 18 chronic conditions in women.**

(continued from previous page)

| First disease | Second disease | n (% prevalence) | Additional comorbidity | Polypharmacy | High-risk medicines | Medicines with anticholinergic activity |
| --- | --- | --- | --- | --- | --- | --- |
| Rheumatoid arthritis | Hypertension | 5 787 (0.33) | 2 686 (46.41) | 1 551 (26.8) | 2 717 (46.95) | 1 415 (24.45) |
|  | Osteoarthritis | 2 386 (0.14) | 1 135 (47.57) | 435 (18.23) | 947 (39.69) | 536 (22.46) |
|  | Depression | 1 998 (0.12) | 906 (45.35) | 373 (18.67) | 750 (37.54) | 515 (25.78) |
| Stroke | Hypertension | 4 192 (0.24) | 2 225 (53.08) | 1 265 (30.18) | 2 160 (51.53) | 864 (20.61) |
|  | Depression | 1 282 (0.07) | 617 (48.13) | 320 (24.96) | 602 (46.96) | 367 (28.63) |
|  | Osteoarthritis | 1 178 (0.07) | 656 (55.69) | 320 (27.16) | 598 (50.76) | 260 (22.07) |
| HIV/AIDS | Hypertension | 736 (0.04) | 268 (36.41) | 53 (7.20) | 147 (19.97) | 105 (14.27) |
|  | Depression | 696 (0.04) | 341 (48.99) | 47 (6.75) | 155 (22.27) | 130 (18.68) |
|  | Mental health disorders | 486 (0.03) | 210 (43.21) | 23 (4.73) | 96 (19.75) | 78 (16.05) |
| Chronic kidney disease | Hypertension | 2 361 (0.14) | 946 (40.07) | 947 (40.11) | 1 306 (55.32) | 695 (29.44) |
|  | Diabetes mellitus | 839 (0.05) | 350 (41.72) | 593 (70.68) | 689 (82.12) | 350 (41.72) |
|  | Osteoarthritis | 781 (0.05) | 358 (45.84) | 358 (45.84) | 458 (58.64) | 272 (34.83) |
| Heart failure | Hypertension | 1 956 (0.11) | 1 026 (52.45) | 866 (44.27) | 1 245 (63.65) | 552 (28.22) |
|  | Diabetes mellitus | 867 (0.05) | 424 (48.90) | 514 (59.28) | 652 (75.20) | 283 (32.64) |
|  | Osteoarthritis | 846 (0.05) | 436 (51.54) | 340 (40.19) | 537 (63.48) | 254 (30.02) |
| Dementia | Hypertension | 1 939 (0.11) | 751 (38.73) | 732 (37.75) | 997 (51.42) | 564 (29.09) |
|  | Osteoarthritis | 747 (0.04) | 330 (44.18) | 211 (28.25) | 325 (43.51) | 194 (25.97) |
|  | Diabetes mellitus | 623 (0.04) | 253 (40.61) | 304 (48.80) | 391 (62.76) | 184 (29.53) |
| Parkinson's disease | Hypertension | 830 (0.05) | 415 (50.00) | 338 (40.72) | 374 (45.06) | 242 (29.16) |
|  | Osteoarthritis | 470 (0.03) | 274 (58.30) | 184 (39.15) | 216 (45.96) | 148 (31.49) |
|  | Depression | 391 (0.02) | 200 (51.15) | 133 (34.02) | 169 (43.22) | 150 (38.36) |
| Liver disease | Hypertension | 636 (0.04) | 319 (50.16) | 133 (20.91) | 227 (35.69) | 116 (18.24) |
|  | Osteoarthritis | 317 (0.02) | 169 (53.31) | 48 (15.14) | 120 (37.85) | 58 (18.30) |
|  | Osteoporosis | 259 (0.01) | 146 (56.37) | 51 (19.69) | 86 (33.20) | 38 (14.67) |

Data are n (%), unless stated. ^*^n (% prevalence): Prevalence within multimorbidity population (≥2 chronic conditions). All other percentages indicate the proportion of people within that trajectory presenting the corresponding feature. ^†^Additional comorbidity: ≥1 further chronic condition (third or more). ^‡^Polypharmacy: ≥5 concurrent medicines for ≥6 months (≤30-day gaps). ^§^High-risk medicine: ≥1 medicine classified as high-risk for chronic patients. ^▲^Anticholinergic activity: ≥1 medicine with anticholinergic activity.

**Table S14: Most common trajectories of multimorbidity onset originating from each of the 18 chronic conditions in men.**

| First disease | Second disease | n (% prevalence)^*^ | Additional comorbidity^†^ | Polypharmacy^‡^ | High-risk medicines^§^ | Medicines with anticholinergic activity^▲^ |
| --- | --- | --- | --- | --- | --- | --- |
| Hypertension | Diabetes mellitus | 111 575 (9.35) | 37 507 (33.62) | 28 191 (25.27) | 57 814 (51.82) | 10 675 (9.57) |
|  | Osteoarthritis | 67 081 (5.62) | 25 624 (38.20) | 12 555 (18.72) | 24 173 (36.04) | 8 299 (12.37) |
|  | Malignant neoplasms | 46 203 (3.87) | 16 231 (35.13) | 7 357 (15.92) | 14 583 (31.56) | 4 817 (10.43) |
| Asthma | Hypertension | 27 442 (2.30) | 6 790 (24.74) | 2 120 (7.73) | 4 323 (15.75) | 2 217 (8.08) |
|  | Mental health disorders | 13 953 (1.17) | 1 990 (14.26) | 258 (1.85) | 1 099 (7.88) | 962 (6.89) |
|  | Osteoarthritis | 12 767 (1.07) | 2 704 (21.18) | 525 (4.11) | 1 215 (9.52) | 704 (5.51) |
| Osteoarthritis | Hypertension | 42 325 (3.55) | 14 559 (34.4) | 6 521 (15.41) | 13 300 (31.42) | 4 705 (11.12) |
|  | Diabetes mellitus | 9 701 (0.81) | 4 722 (48.68) | 2 381 (24.54) | 5 420 (55.87) | 1 198 (12.35) |
|  | Malignant neoplasms | 7 117 (0.60) | 2 868 (40.30) | 901 (12.66) | 2 110 (29.65) | 801 (11.25) |
| Mental health disorders | Hypertension | 20 662 (1.73) | 4 762 (23.05) | 1 861 (9.01) | 5111 (24.74) | 3 444 (16.67) |
|  | Depression | 9 260 (0.78) | 1 939 (20.94) | 682 (7.37) | 2710 (29.27) | 2 230 (24.08) |
|  | Asthma | 6 995 (0.59) | 1 028 (14.70) | 214 (3.06) | 800 (11.44) | 656 (9.38) |
| Depression | Hypertension | 23 516 (1.97) | 7 362 (31.31) | 2 929 (12.46) | 6 692 (28.46) | 4 203 (17.87) |
|  | Mental health disorders | 7 037 (0.59) | 1 764 (25.07) | 417 (5.93) | 1 680 (23.87) | 1 418 (20.15) |
|  | Osteoarthritis | 6 556 (0.55) | 2 601 (39.67) | 758 (11.56) | 1 900 (28.98) | 1 288 (19.65) |
| Diabetes mellitus | Hypertension | 88 627 (7.43) | 30 464 (34.37) | 26 044 (29.39) | 51 285 (57.87) | 8 724 (9.84) |
|  | Osteoarthritis | 13 905 (1.16) | 6 944 (49.94) | 4 018 (28.90) | 8 394 (60.37) | 1 701 (12.23) |
|  | Ischaemic heart disease | 11 028 (0.92) | 5 210 (47.24) | 4 787 (43.41) | 7 153 (64.86) | 1 559 (14.14) |
| Malignant neoplasms | Hypertension | 29 490 (2.47) | 10 702 (36.29) | 4 479 (15.19) | 8 896 (30.17) | 3 002 (10.18) |
|  | Diabetes mellitus | 7 987 (0.67) | 4 043 (50.62) | 1 966 (24.61) | 4 330 (54.21) | 949 (11.88) |
|  | Osteoarthritis | 7 155 (0.60) | 3 193 (44.63) | 923 (12.90) | 2 175 (30.40) | 794 (11.10) |
| Osteoporosis | Hypertension | 1 648 (0.14) | 753 (45.69) | 340 (20.63) | 616 (37.38) | 245 (14.87) |
|  | Osteoarthritis | 783 (0.07) | 420 (53.64) | 138 (17.62) | 287 (36.65) | 142 (18.14) |
|  | Malignant neoplasms | 390 (0.03) | 185 (47.44) | 58 (14.87) | 123 (31.54) | 65 (16.67) |
| Ischaemic heart disease | Hypertension | 23 185 (1.94) | 11 031 (47.58) | 7 096 (30.61) | 11 909 (51.37) | 3 206 (13.83) |
|  | Diabetes mellitus | 11 867 (0.99) | 5 570 (46.94) | 4 868 (41.02) | 7 387 (62.25) | 1646 (13.87) |
|  | Osteoarthritis | 6 071 (0.51) | 3 005 (49.50) | 1 850 (30.47) | 3 246 (53.47) | 976 (16.08) |
| COPD | Hypertension | 17 486 (1.46) | 7 882 (45.08) | 4 474 (25.59) | 6 407 (36.64) | 2 827 (16.17) |
|  | Diabetes mellitus | 5 775 (0.48) | 3 034 (52.54) | 1 979 (34.27) | 3 279 (56.78) | 1 007 (17.44) |
|  | Osteoarthritis | 4 571 (0.38) | 2 525 (55.24) | 1 157 (25.31) | 1 764 (38.59) | 880 (19.25) |

(Table S14 continues on next page)

**Table S14: Most common trajectories of multimorbidity onset originating from each of the 18 chronic conditions in men.**

(continued from previous page)

| First disease | Second disease | n (% prevalence) | Additional comorbidity | Polypharmacy | High-risk medicines | Medicines with anticholinergic activity |
| --- | --- | --- | --- | --- | --- | --- |
| Rheumatoid arthritis | Hypertension | 2 403 (0.20) | 934 (38.87) | 533 (22.18) | 1 007 (41.91) | 408 (16.98) |
|  | Osteoarthritis | 721 (0.06) | 316 (43.83) | 131 (18.17) | 311 (43.13) | 128 (17.75) |
|  | Diabetes mellitus | 566 (0.05) | 281 (49.65) | 184 (32.51) | 343 (60.60) | 111 (19.61) |
| Stroke | Hypertension | 6 961 (0.58) | 3 026 (43.47) | 1 887 (27.11) | 3 571 (51.30) | 1 005 (14.44) |
|  | Diabetes mellitus | 1 841 (0.15) | 971 (52.74) | 757 (41.12) | 1 206 (65.51) | 309 (16.78) |
|  | Depression | 1 051 (0.09) | 489 (46.53) | 302 (28.73) | 541 (51.47) | 263 (25.02) |
| HIV/AIDS | Hypertension | 2 302 (0.19) | 744 (32.32) | 131 (5.69) | 394 (17.12) | 190 (8.25) |
|  | Depression | 1 517 (0.13) | 587 (38.69) | 87 (5.74) | 284 (18.72) | 220 (14.5) |
|  | Mental health disorders | 1 438 (0.12) | 486 (33.80) | 55 (3.82) | 237 (16.48) | 193 (13.42) |
| Chronic kidney disease | Hypertension | 3 229 (0.27) | 1 173 (36.33) | 970 (30.04) | 1 456 (45.09) | 546 (16.91) |
|  | Diabetes mellitus | 1 132 (0.09) | 433 (38.25) | 666 (58.83) | 854 (75.44) | 257 (22.70) |
|  | Osteoarthritis | 657 (0.06) | 244 (37.14) | 202 (30.75) | 302 (45.97) | 128 (19.48) |
| Heart failure | Hypertension | 2 007 (0.17) | 863 (43.00) | 675 (33.63) | 1 071 (53.36) | 298 (14.85) |
|  | Diabetes mellitus | 996 (0.08) | 425 (42.67) | 553 (55.52) | 743 (74.60) | 193 (19.38) |
|  | COPD | 644 (0.05) | 274 (42.55) | 293 (45.50) | 399 (61.96) | 120 (18.63) |
| Dementia | Hypertension | 811 (0.07) | 293 (36.13) | 294 (36.25) | 399 (49.2) | 205 (25.28) |
|  | Diabetes mellitus | 327 (0.03) | 128 (39.14) | 145 (44.34) | 203 (62.08) | 96 (29.36) |
|  | Osteoarthritis | 267 (0.02) | 116 (43.45) | 82 (30.71) | 131 (49.06) | 72 (26.97) |
| Parkinson's disease | Hypertension | 930 (0.08) | 356 (38.28) | 340 (36.56) | 378 (40.65) | 242 (26.02) |
|  | Osteoarthritis | 356 (0.03) | 161 (45.22) | 129 (36.24) | 148 (41.57) | 92 (25.84) |
|  | Diabetes mellitus | 303 (0.03) | 140 (46.20) | 155 (51.16) | 196 (64.69) | 95 (31.35) |
| Liver disease | Hypertension | 852 (0.07) | 360 (42.25) | 131 (15.38) | 311 (36.50) | 110 (12.91) |
|  | Diabetes mellitus | 551 (0.05) | 292 (52.99) | 124 (22.5) | 302 (54.81) | 58 (10.53) |
|  | Malignant neoplasms | 247 (0.02) | 106 (42.91) | 38 (15.38) | 103 (41.70) | 36 (14.57) |

Data are n (%), unless stated. ^*^n (% prevalence): Prevalence within multimorbidity population (≥2 chronic conditions). All other percentages indicate the proportion of people within that trajectory presenting the corresponding feature. ^†^Additional comorbidity: ≥1 further chronic condition (third or more). ^‡^Polypharmacy: ≥5 concurrent medicines for ≥6 months (≤30-day gaps). ^§^High-risk medicine: ≥1 medicine classified as high-risk for chronic patients. ^▲^Anticholinergic activity: ≥1 medicine with anticholinergic activity.

**Table S15: All-cause mortality in 2021 in the general population and among those with multimorbidity or multisystem multimorbidity, stratified by sex.**

| **Population group** | **Outcome** | | **All** | **Women** | **Men** | ***p* values** |
| --- | --- | --- | --- | --- | --- | --- |
| **General population** | | Mortality | 145 747 (0.84) | 71 949 (0.81) | 73 798 (0.87) | <0.0001 |
|  | | Mortality <50 years | 4 567 (0.03) | 1 686 (0.02) | 2 881 (0.03) | <0.0001 |
|  | | Mortality <65 years | 19 409 (0.11) | 6 632 (0.07) | 12 777 (0.15) | <0.0001 |
| **With multimorbidity** | | Mortality | 100 374 (3.43) | 52 842 (3.05) | 47 532 (3.97) | <0.0001 |
|  | | Mortality <50 years | 850 (0.03) | 361 (0.02) | 489 (0.04) | <0.0001 |
|  | | Mortality <65 years | 7 090 (0.24) | 2 648 (0.15) | 4 442 (0.37) | <0.0001 |
| **With multisystem multimorbidity** | | Mortality | 88 986 (3.65) | 48 379 (3.17) | 40 607 (4.46) | <0.0001 |
|  | | Mortality <50 years | 701 (0.03) | 318 (0.02) | 383 (0.04) | <0.0001 |
|  | | Mortality <65 years | 5 974 (0.25) | 2 412 (0.16) | 3 562 (0.39) | <0.0001 |

Values in parentheses indicate the percentage of mortality within each population subgroup.

**Table S16: Performance metrics for gradient boosting models predicting advanced, multisystem, and complex multimorbidity, polypharmacy, and supply of high-risk or anticholinergic-activity medicines.**

| **Outcome** | **Accuracy** | **F1 score** | **Recall** | **Precision** | **AUC** |
| --- | --- | --- | --- | --- | --- |
| Advanced multimorbidity^*^ | 0.83 | 0.46 | 0.93 | 0.30 | 0.94 |
| Multisystem multimorbidity^†^ | 0.81 | 0.58 | 0.95 | 0.42 | 0.93 |
| Complex multimorbidity^‡^ | 0.85 | 0.27 | 0.94 | 0.16 | 0.95 |
| Polypharmacy^**^ | 0.92 | 0.56 | 0.92 | 0.40 | 0.96 |
| Supply of high-risk medicine^††^ | 0.82 | 0.53 | 0.79 | 0.39 | 0.89 |
| Supply of medicines with anticholinergic activity^‡‡^ | 0.90 | 0.52 | 0.84 | 0.37 | 0.92 |

*Accuracy* = proportion of all predictions that are correct; *F1 score* = harmonic mean of precision and recall; *Recall* = proportion of true positives correctly identified; *Precision* = proportion of predicted positives that are correct; *AUC* = area under the receiver operating characteristic curve, measuring overall discrimination ability. ^*^Advanced multimorbidity: ≥3 chronic conditions. ^†^Multisystem multimorbidity: Conditions affecting ≥2 organ systems. ^‡^Complex multimorbidity: Conditions affecting ≥3 organ systems. ^**^Polypharmacy: ≥5 concurrent medicines for ≥6 months (≤30-day gaps). ^††^High-risk medicine: ≥1 medicine classified as high-risk for chronic patients. ^‡‡^Anticholinergic activity: ≥1 medicine with known anticholinergic properties.

**Table S17: Optimal age at first recorded diagnosis cut-off points for adverse outcomes, derived from ROC curves using the Youden index.**

| **Outcome** | **Optimal cut-off age (years)** | **Youden index** | **Accuracy** | **Sensitivity** | **Specificity** |
| --- | --- | --- | --- | --- | --- |
| Advanced multimorbidity | 53 | 0.37 | 0.64 | 0.76 | 0.61 |
| Multisystem multimorbidity | 52 | 0.31 | 0.65 | 0.68 | 0.63 |
| Complex multimorbidity | 54 | 0.35 | 0.62 | 0.75 | 0.60 |
| Polypharmacy | 56 | 0.41 | 0.67 | 0.76 | 0.65 |
| Supply of high-risk medicines | 52 | 0.33 | 0.64 | 0.73 | 0.61 |
| Supply of medicines with anticholinergic activity | 53 | 0.23 | 0.58 | 0.66 | 0.57 |

The optimal cut-off points for the age at first recorded diagnosis for each outcome measure were derived from receiver operating characteristic (ROC) curves. The graphical technique was used to assess for age thresholds to model the occurrence of an outcome. The ROC curves were obtained by calculating the sensitivity and specificity of the outcome measure at every possible cut-off point, and plotting sensitivity against 1 -specificity. The Youden index method was used to establish the optimal cut-off points from the ROC curves (i.e., the age where the optimal sensitivity and specificity are achieved). Cut-off ages correspond to the point on the ROC curve maximising the Youden index (sensitivity + specificity − 1). The analyses were performed using the ‘cutpointr’ R software package.

**References**

Youden WJ. Index for rating diagnostic tests. Cancer 1950; 3: 32–5.

“Cutpointr” R software package, https://cran.r-project.org/web/packages/cutpointr/vignettes/cutpointr.html (accessed Aug 11, 2025).

Thiele C, Hirschfeld G (2021). Cutpointr: Improved Estimation and Validation of Optimal Cutpoints in R. Journal of Statistical Software, 98 (11), 1–27. doi:10.18637/jss.v098.i11.
